# Design of LED lighting system using solar powered PV cells for a proposed business complex

**DOI:** 10.1038/s41598-022-17353-2

**Published:** 2022-08-02

**Authors:** Sayan Kumar Nag, Tarun Kumar Gangopadhyay

**Affiliations:** Department of Electrical Engineering, Techno Main Salt Lake, Kolkata, West Bengal India

**Keywords:** Solar energy, Electrical and electronic engineering

## Abstract

The paper outlines the concepts and design of an upcoming stand-alone solar photovoltaic system to supply the energy needs of a new proposed business complex. The purpose of this study is to develop a prediction method for the use of solar energy for commercial purposes. Firstly, the annual energy demand for the illumination and utilities in a business complex is calculated. LED lights are considered in the complex for the illumination to minimize the cost of energy. Based on the load estimation, the number of solar panels are predicted as 6097, to generate the power for the proposed area. Secondly, the important part of the design in the estimation of solar radiation and optimal tilt angle of a photovoltaic panel has been calculated for the maximum energy harvest. In this case the optimal tilt angle is 49.34°. The installation of PV panels for optimal and feasible operation is also predicted. The calculated parameters are used in a simulation with a software to test their practicality in the business complex. The technique is used to determine the amount of energy produced and system’s performance ratio. A cost estimate is also provided for the solar PV system. In the end, an analysis of these simulations and estimations is presented.

## Introduction

A solar photovoltaic power plant converts sunlight into electricity by using photovoltaic cells, also known as PV or solar cells^1^. Alloys of silicon are used to make these cells^2^. Solar energy is directly converted into electricity by photovoltaic cells. They work according to the principle of photovoltaics^3^. Photons are absorbed by certain elements when exposed to light, which releases free electrons. The photoelectric effect is the term used to describe this phenomenon^4^. The photovoltaic effect is the process of producing direct current electricity using the principle of photoelectric effect^5^.

Based on the principle of photovoltaic effect, solar cells or photovoltaic cells are created^6^. Sunlight is converted by them into direct current (DC) electricity^7^. But the amount of electricity generated by one solar cell is not adequate^8^. Hence, solar modules or solar panels are comprised of several solar cells mounted onto a support frame and electrically connected to one another^7^.

In general, solar panels are available in a range of sizes and power outputs, ranging from several hundred watts up to several kilowatts^9^. While panels or modules deliver power at a certain voltage, the current they produce is determined by the intensity of the incident light^10^. In most cases, however, solar power systems are also equipped with inverters to provide AC power^11^.

India consumes about 6% of global energy^12^. India has an installed generation capacity of 365 GW of which 55.8% is coal powered, 13.7% is hydroelectricity, 10.1% is wind powered, solar PV is of 8.8%, 6.8% is of natural gas, bio energy and waste is of 2.7%, 2% is nuclear powered, 0.1% is oil powered^13^.

Solar energy makes up of about 8.8% of the electricity generated^14^. This is because solar power plants cannot be deployed in regions having inconsistent sunlight or because of the large surface area required to capture solar energy^15^. However, in solar-rich nations and regions electricity produced from solar panels is cheaper than electricity produced commercially^16^. In the recent years, use of solar energy has seen an exponential rise in India^17^. Advances in solar panel efficiency are expected to make solar energy more readily available for use in home and office buildings^18^.

The present project is aimed to focus on the study of an energy efficient illumination and utilities system using LED lamps, for a business complex. Utilizing systems like light-emitting diode (LED) instead of traditional lamps can reduce electricity consumption^19^. The scope of the work is to design an effective solar photovoltaic system which would meet the complete energy demand of a proposed business complex without consuming conventional energy supply.

**Figure 1:**
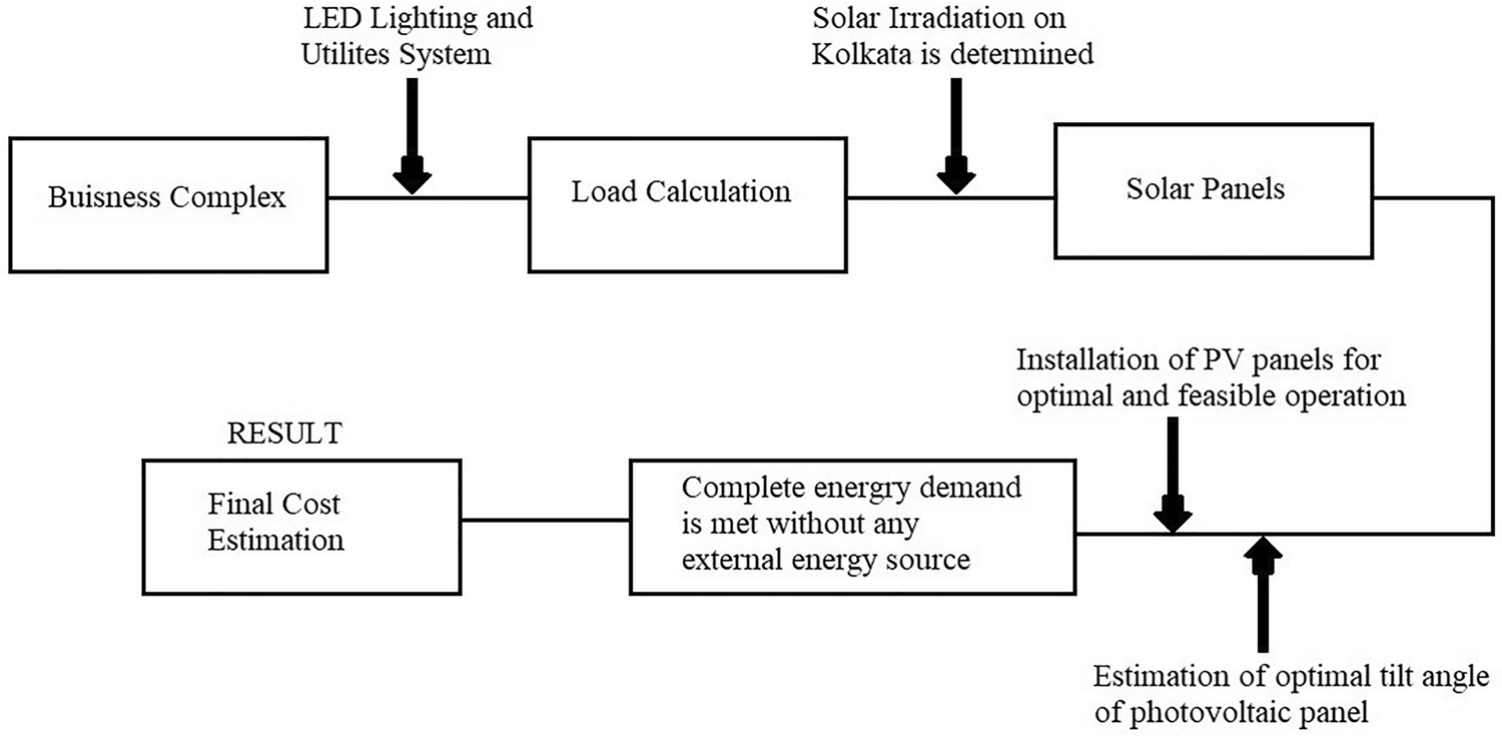
Block diagram of the design concept on present project.

A block diagram of the tentative process is shown in Fig. 1.

## Solar photovoltaic power plant types

**Figure 2:**
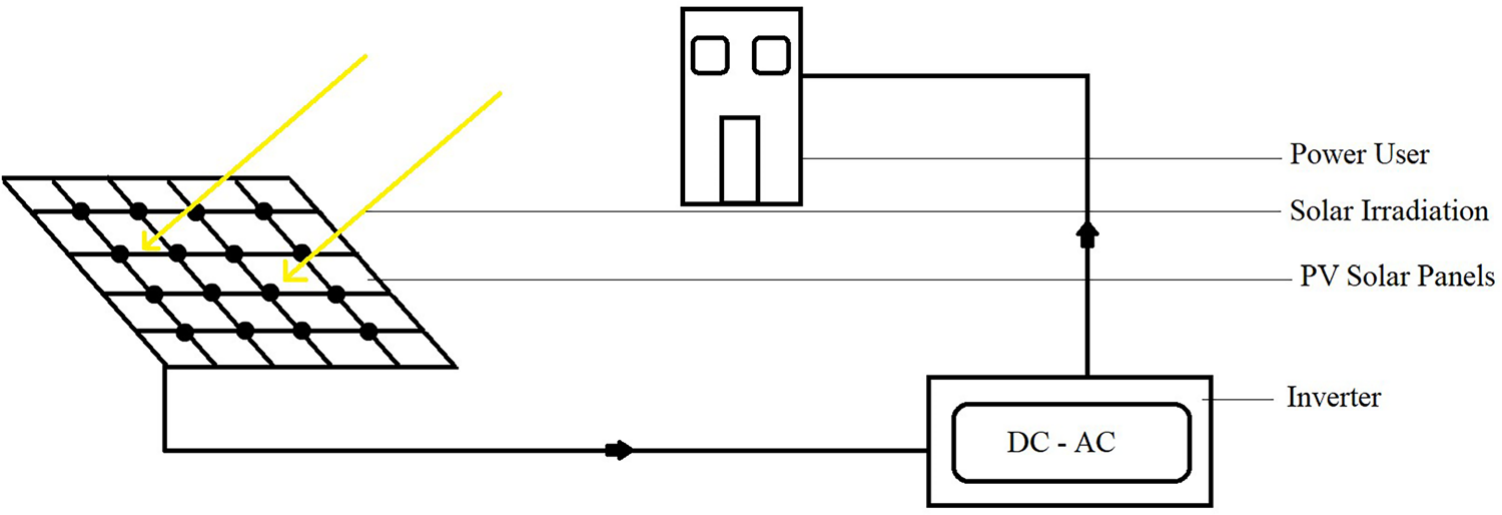
PV direct system^22^.

**Figure 3:**
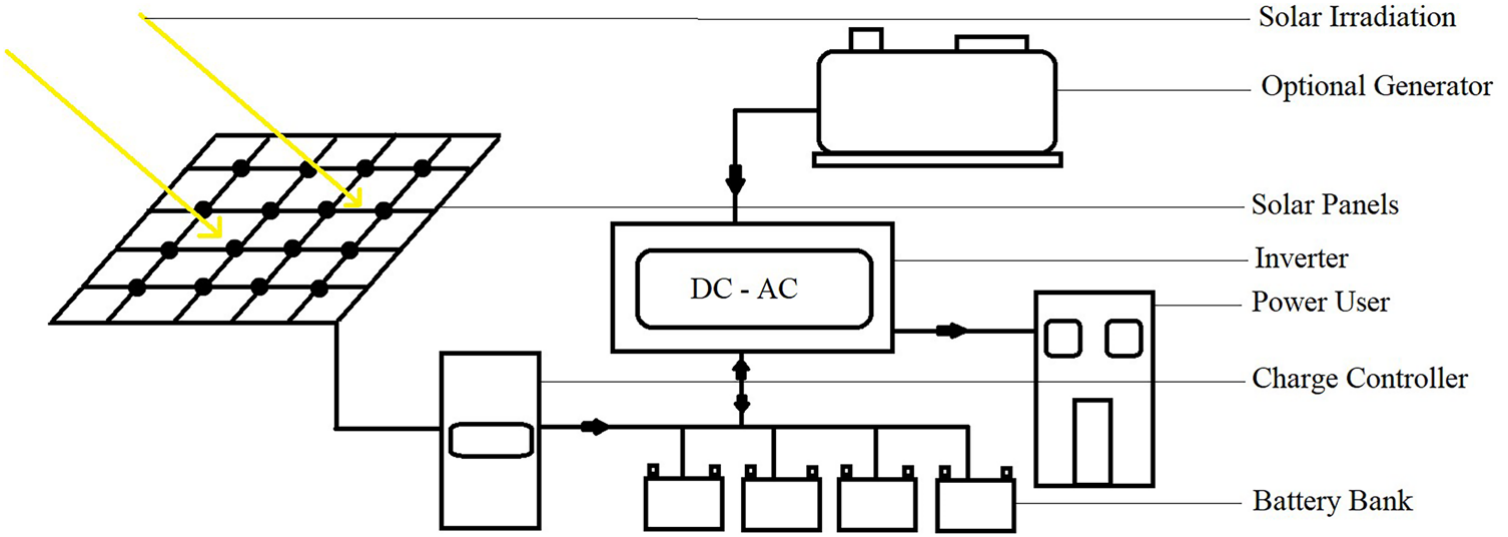
Off-grid system^23^.

**Figure 4:**
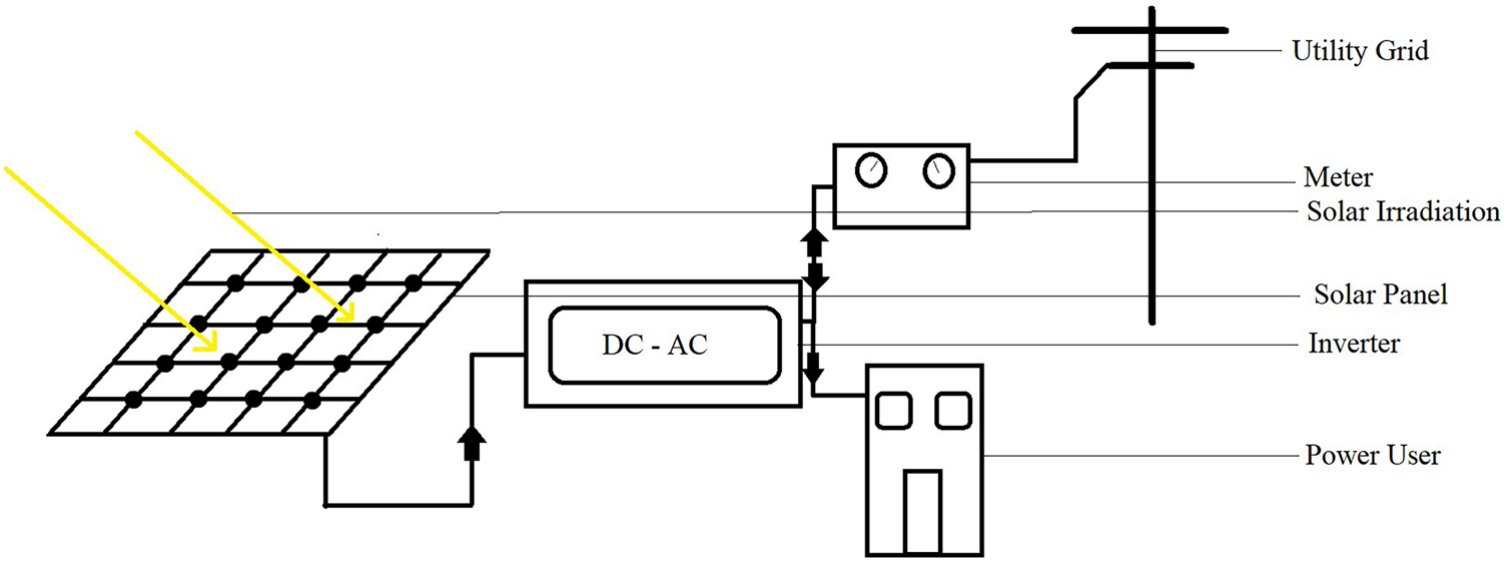
Grid connected system^23^.

Depending on the power requirements, several solar modules are electrically connected to form a PV array and achieve more power^20^. There are different types of PV systems depending on their application:

i. PV direct systems
  This system only supplies the load when the sun is shining. There is no battery as the generated power is not stored^21^. A diagram of PV direct system is shown in Fig. 2.
ii. Off-grid Systems
  Systems of this type are usually used in locations where the power of the grid is not available or reliable. There is no electrical grid connection for off-grid solar power systems. It has a solar panel array, storage battery and inverter circuit^21^. A diagram of off-grid system is shown in Fig. 3.
iii. Grid connected systems
  Taking advantage of the grid connection, additional energy can be obtained from the grid for these solar power systems. It may or may not be backed by batteries^21^. A diagram of grid connected system is shown in Fig. 4.

## Construction of a solar PV power plant

The process of building a solar PV power plant is a complex effort that requires considerable time cost and expertise.

It can be divided into following stages:

i. Identification of the location.
ii. Determination of the grid connection point.
iii. Pre-construction documentation and negotiations.
iv. Building the infrastructure i.e., roads, fence, security, etc.
v. Equipment and logistics purchase.
vi. Installation of supporting structures.
vii. Installation of the transformer substation.
viii. Connection with the grid.
ix. System setup monitoring^24^.

## Scheme of the present research

### A layout of proposed area of illumination in the business complex

**Figure 5:**
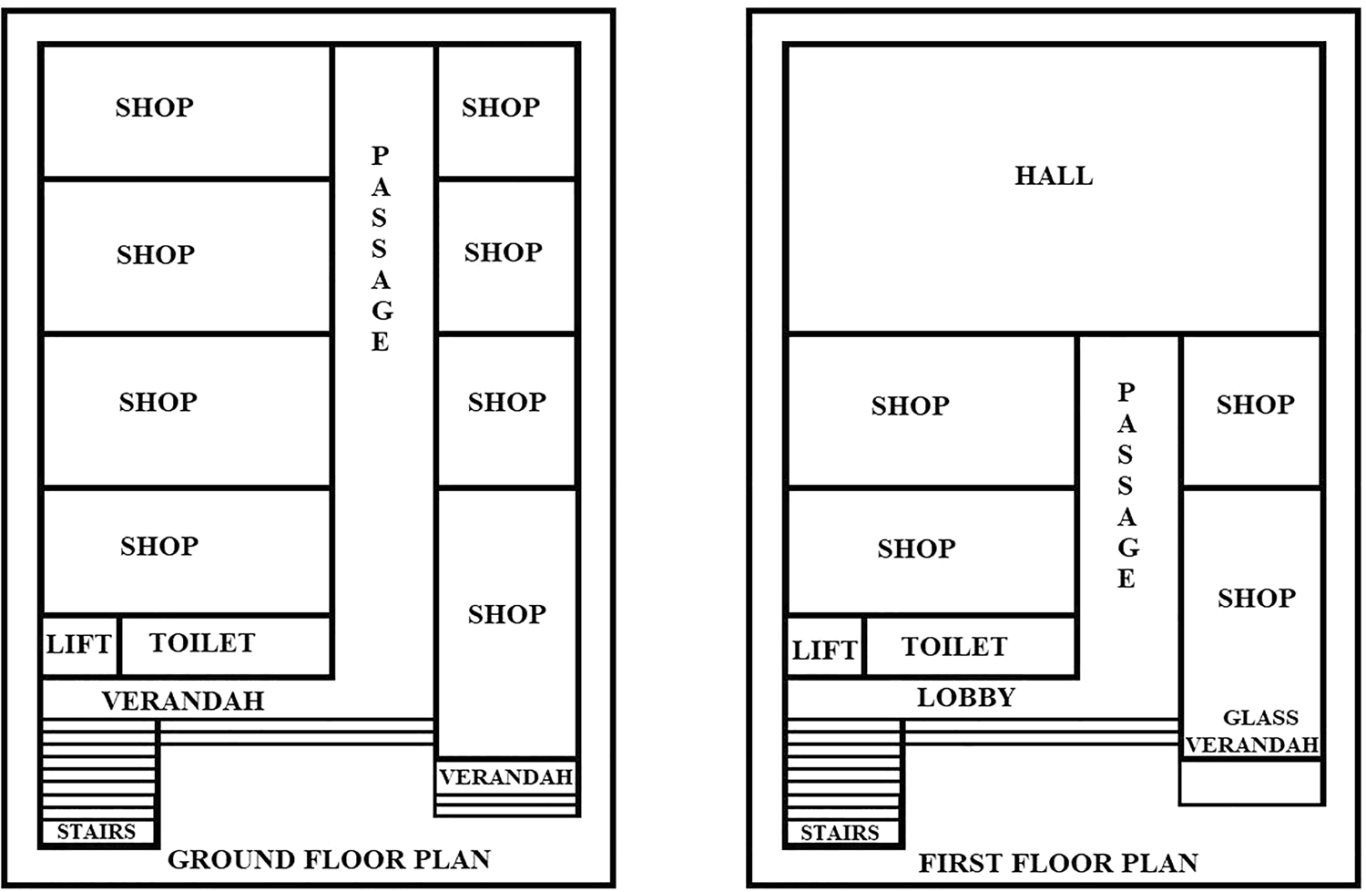
Layout (proposed area of illumination) of the business complex^25^.

The proposed layout of the building or structure is shown in Fig. 5. It is established according to the foundation plan drawings and specifications provided by the engineer or architect.

Presently, building design focuses on maximizing sustainability, environmental impact and energy efficiency. All these factors have a direct effect on the power distribution in the building. In practice, electrical engineers must work with architects and mechanical engineers to achieve the most accurate estimates of demand.

### Calculation of loads for illumination and appliances used in this business complex

The projected electricity demand identifies the building’s requirements in terms of power supply and distribution. Therefore, an accurate estimate of the amount of power required for a building to function successfully is an essential step in planning and designing a system. Appliances used in the business complex with their power rating is shown in Table 1. Finally, total load of the proposed business complex is shown in Table 2.

**Table 1:** Appliances used in the Business Complex with their power rating^26,27^.

Sl. no	Appliance	Quantity	Individual power rating (Watt)	Hours used
1	LED bulb—40 Watt equivalent	21	10	14
2	LED bulb—60 Watt equivalent	132	13	14
3	LED bulb—75 Watt equivalent	29	18	14
4	LED bulb—100 Watt Equivalent	3	23	6
5	Blender	3	500	10
6	Coffee machine	2	1000	10
7	Freezer—Chest—15 cu. ft	2	1080	24
8	Garbage disposal	1	450	2
9	Kettle—electric	1	1200	4
10	Microwave	2	1000	10
11	TV—LCD	2	150	14
12	Cable box	2	35	14
13	Central air conditioner—24,000 BTU NA	2	3800	14
14	Vacuum	4	1000	2
15	Desktop computer (standard)	13	200	14
16	Printer	13	100	14
17	Elevator	1	7631 (kWh)	14
18	Box fan	1	200	14
19	Furnace fan blower	8	800	14
20	Smart phone—recharge	26	6	14

**Table 2:** Total load of the proposed business complex.

Sl. no	Total load	Value
1	Total load of the business complex in a day	5,22,824 Watt-hours/day
		522.824 Kilowatt-hours/day
2	Total load of the business complex in a year	5,22,824 Watt-hours × 365
		19,08,30,760 Watt-hours/year
		1,90,830.760 Kilowatt-hours/year
		190.831 Megawatt-hours/year

### The required value of the annual solar incidence of the area

Solar PV systems must be designed based on how much sunlight can be harnessed at any given time and place. Solar radiation (or radiation) and solar isolation are the two most predominant methods of solar radiation. Solar radiance is described as the instantaneous power density in the units of kW/m^2^. Solar radiance varies, from 0 kW/m^2^ at night up to 1 kW/m^2^ during the daytime^27^. Location and local weather are also crucial factors affecting solar radiance. Pyranometers (for measuring global radiation) and pyrheliometers (for measuring direct radiation) are used for the measurements. These data have been collected for over two decades at a well-established location^28^.

**Figure 6:**
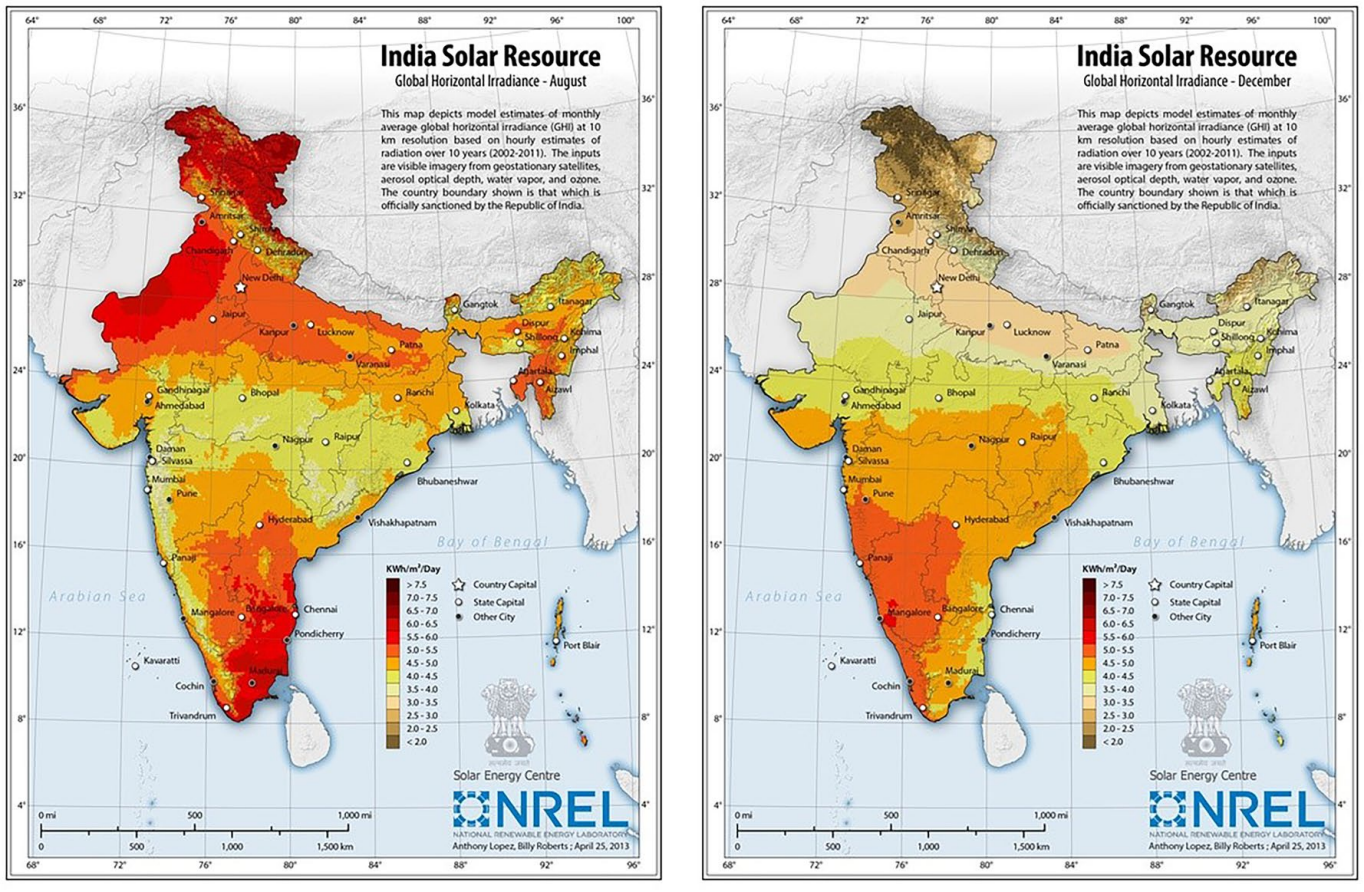
Solar radiation map of India^29^.

Sunshine recorders are also an inexpensive and less accurate method of measuring solar radiation. Sunshine recorders (also known as Campbell-Stokes recorders) measure how many hours during the day sunlight is above a certain level (usually 200 mW/cm^2^)^27^. Data collected in this manner can be used to calculate insolation as a function of the hours of sunshine measured with a value calculated using several correction factors^28^. Finally, cloud cover data collected from existing satellite images can be used to estimate solar insolation as illustrated in Fig. 6. A solar radiation map of India is shown in Fig. 6. ^29^.

### Designing of the PV system

From Fig. 5, Solar Irradiance in Kolkata is 3.5 to 4.0 kWh/m^2^/day. Annual full sun hours in Kolkata is 3.5 × 365 = 1227.5 kWh/m^2^/year. Efficiency of PV cell considering it a polycrystalline cell is 17%^30^.

Therefore,

$$\mathrm{PV\,System\,Size }=\frac{\frac{\mathrm{19,08,30.760}}{1227.5}}{0.17}= 914.4878\mathrm{ kW}$$

No. of modules **=** $\frac{914487.8 }{150}$  = 6096.5853 ≃ 6097 Considering Module power is 150 W for each module^31^.

Therefore, we would require approximately 6097 panels to supply complete power to the proposed business complex.

## Theory of solar angles

### Air mass

Considering the vertical path of the sea surface as unity, the path length of the sun's rays through the atmosphere is described in terms of “Air Mass”^32^.

Alternatively, the air mass “m” is the ratio of the path length of the sun's rays through the atmosphere to the length of the path when the sun's position is directly overhead (i.e., the zenith position)^32^.

Thus, m = $$\frac{\mathrm{The\,length\,of\,the\,path\,transversed\,by\,the\,beam\,radiation}}{\mathrm{Atmospheric\,vertical\,path\,length}}$$

**Figure 7:**
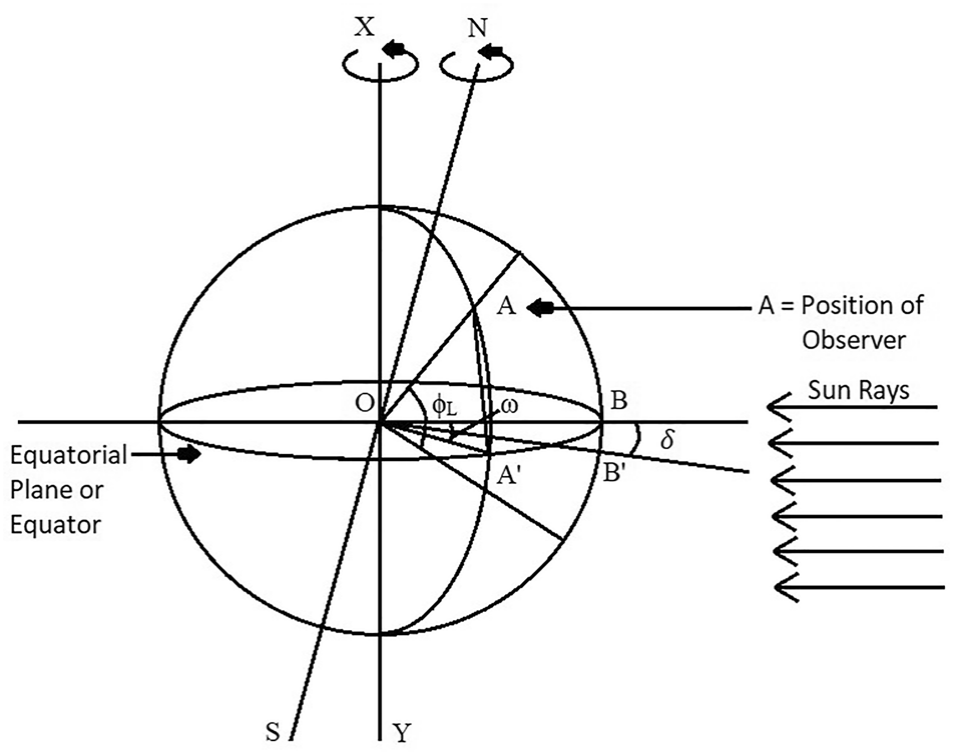
Solar radiation geometry^32^.

Figure 7 shows the solar radiation geometry and the various angles formed which will be explained in next sub-sections.

**Figure 8:**
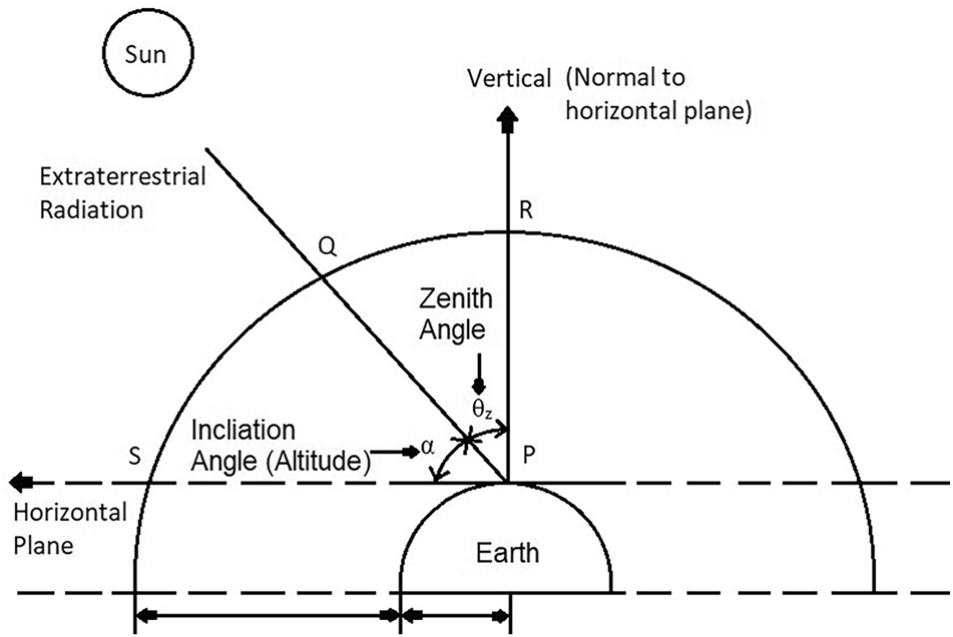
Direction of sun’s ray with respect to atmosphere^32^.

According to Fig. 8,

1 $$ m = \frac{PQ}{{PR}} = {\text{ sec}}\,\theta_{{\text{z}}} = {\text{ cosec}}\,\alpha $$

where, α = Inclination angle or Altitude angle, θ_z_ = Zenith angle.

Therefore,

2 $$ \alpha \, + \theta_{{\text{z}}} = { 9}0^\circ $$

### Zenith angle

It is the angle between the rays of the sun and the vertical plane. It is denoted by “θ_z_”^32^.

According to Fig. 8,

3 $$ \theta_{{\text{z}}} = \angle {\text{QPR }} = \frac{\pi }{2} - \alpha $$

According to Eq. (1), the air mass is equal to the cosecant of the altitude angle (α). Therefore, at sea level m = 1.

m = 1 when the sun is at zenith position (i.e., overhead).

m = sec θ_z_, when m > 3.

m = 0, Just above the Earth’s atmosphere.

### Angle of latitude

Latitude is the angle formed by the radial line connecting a given location to the centre of the Earth. This line is projected onto the equatorial plane to determine the latitude of the vertical space on the surface of the Earth^32^.

From the centre of the earth, it is the number of degrees of angle north–south of the equator^32^. A positive latitude indicates the northern hemisphere and a negative one indicates the southern hemisphere. It is denoted by ϕ_L_.

**Figure 9:**
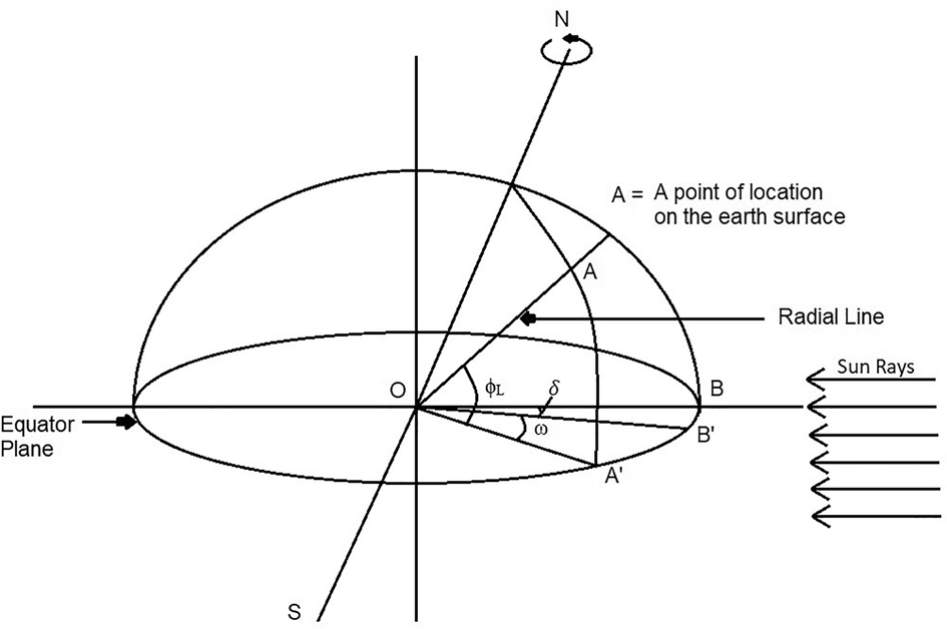
Latitude of location (ϕ_L_), Hour angle (ω) and Sum’s declination angle (δ)^32^.

As per Fig. 9, angle of latitude is the angle between the line OA and the projection line OA’, on the equator plane. Point A, depicts the location on the earth’s surface, Point O, depicts the centre of the earth. By convention, the latitude will be + ve for the North Hemisphere.

Therefore, according to Fig. 9, ϕ_L_ is ∠AOA’.

### Declination angle

Declination angle is the angular distance of the sun's rays north (or south) from the Earth's equatorial plane. It is denoted by the symbol δ^32^.

**Figure 10:**
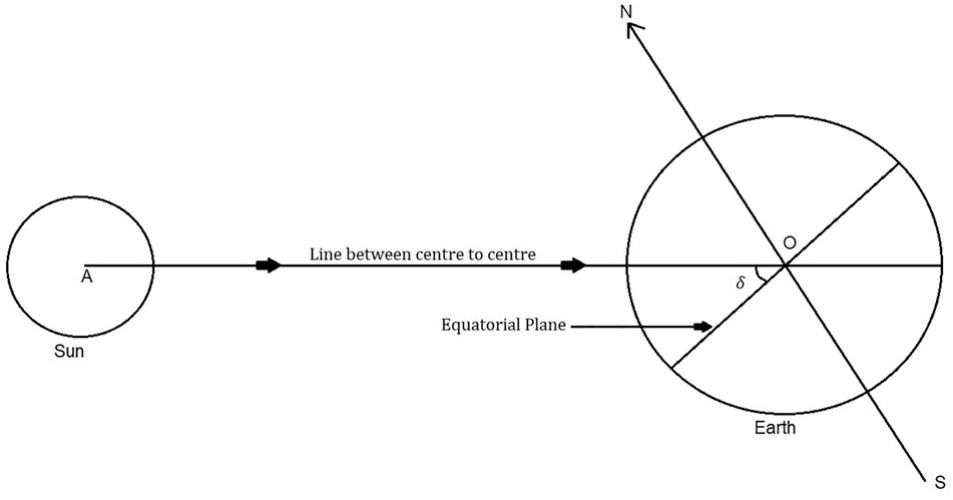
Declination angle (δ)^32^.

It can also be defined as the angle between the line running from the centre of the Sun to the centre of the Earth and the projection of this line on the equatorial plane of the earth^32^. It is shown in Fig. 10.

**Figure 11:**
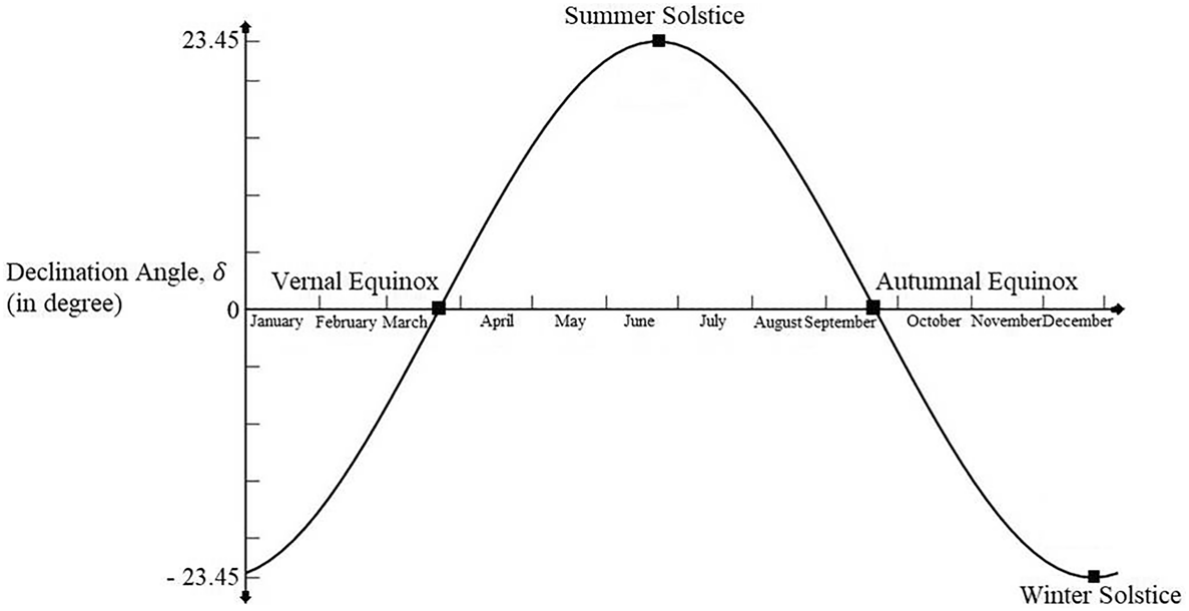
Variation of sun’s declination^33^.

When measured from above the equatorial plane declination angle is positive in the northern hemisphere^32^. This is the direct consequence of the tilt and it would vary between 23.5° on June 22 to − 23.5° on December 22. For minimum or maximum declination, the sun appears in a stand still condition. This condition is called a solstice. During winter solstice, the sun rays would be 23.5° south of earth’s equator i.e., δ is − 23.5°. During summer solstice, the sun rays would be 23.5° north of earth’s equator i.e., δ is 23.5°. The variation of sun’s declination is shown in Fig. 11.

The declination of the angle can be determined from Cooper's approximate equation.

4 $$ \delta \left( {\text{in degree}} \right) \, = { 23}.{45 } \times {\text{sin }}\left[ {\frac{360}{{365}}(284 + n)} \right]({\text{degree)}} $$

where, “n” is the day of the year counted from 1st January.

### Hour angle

The angle at which the Earth must rotate to get the meridian of a particular point (or the observer point) directly in line with the sun's rays is called the hour angle at that moment. It is denoted by ω^32^.

**Figure 12:**
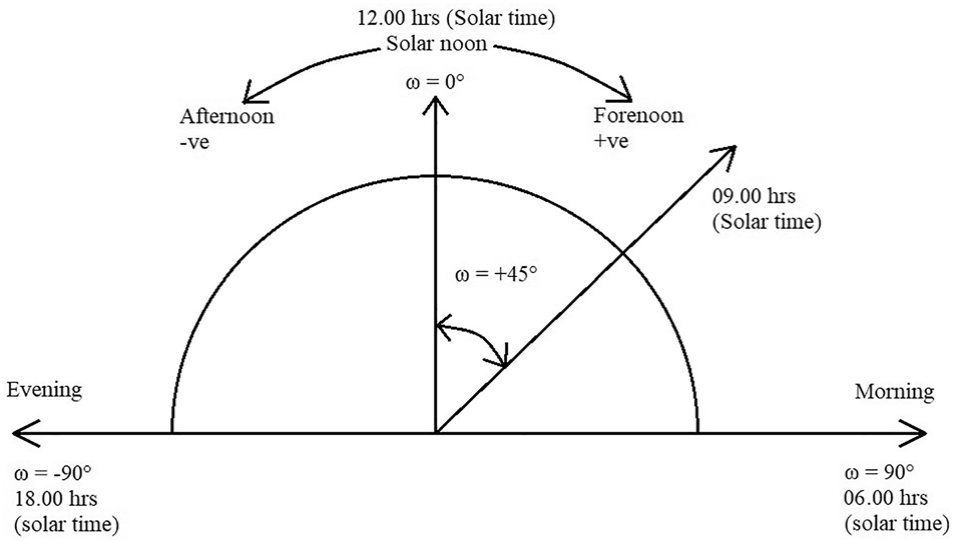
The hour angle (ω)^32^.

In other words, at any moment, it is the angular displacement of the sun towards East or West of local meridian (due to rotation of the earth on its axis)^32^. The Hour angle (ω) is shown in Fig. 12.

As per figure, at 06.00 h. ω is + 90° and at evening, 18.00 h., ω is − 90°.

For example, ω is − 15° at 11:00 a.m., ω is 0° at 12:00 p.m. and ω is 15° at 1:00 p.m. Hence, the difference or addition of 15° at every hour.

On considering Fig. 11, to calculate Hour angle (ω) is the angle measured in the Earth’s equatorial plane, between the projection of OA and the projection of a line from the centre of the sun to the centre of the earth.

Therefore, Considering Fig. 12,

Hour Angle, ω is ∠A’OB’.

### Altitude angle or inclination angle

The altitude angle indicates how high the sun appears in the sky. The angle is measured between the imaginary line between the observer and the sun and the horizontal plane on which the observer is standing^32^. When the sun falls below the horizon, the altitude angle is negative. It is also known as inclination angle and solar elevation angle. It is denoted by “α”.

According to Fig. 8,

α = Inclination angle or Altitude angle = ∠QPS.

**Figure 13:**
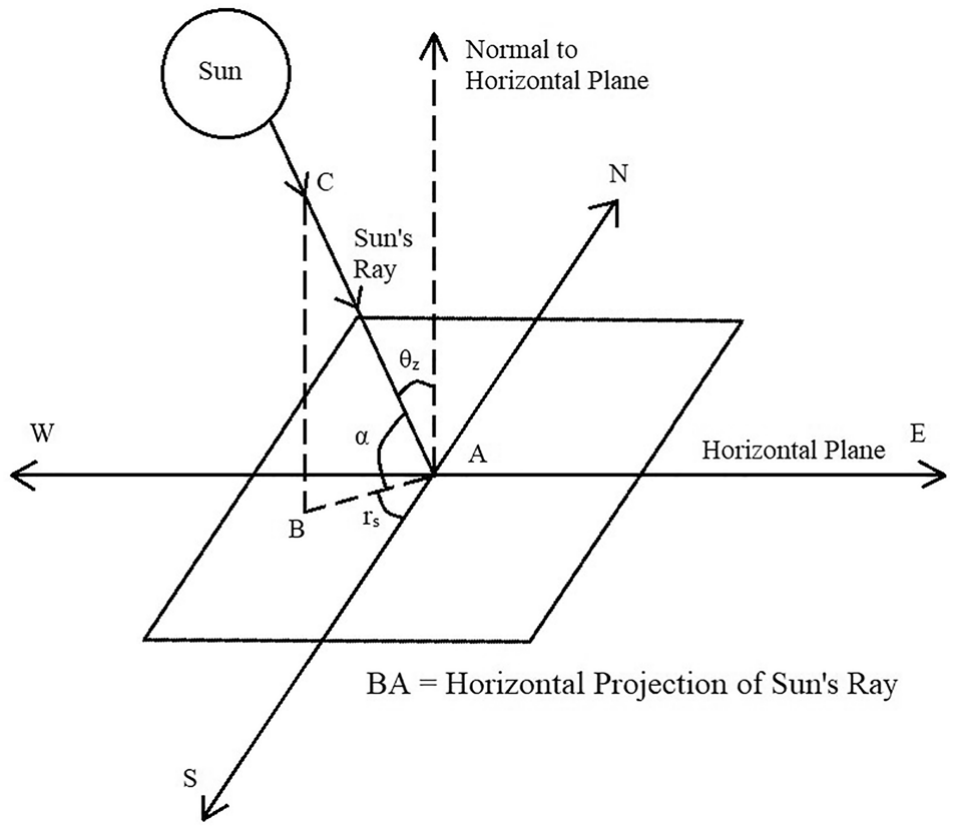
Solar inclination angle (α), Zenith angle(θ_z_) and solar Azimuth angle(r_s_)^32^.

According to Fig. 13,

α = ∠CAB = Inclination angle or Altitude angle.

As (α + θ_z_) = 90° = $$\frac{\uppi }{2}$$

Therefore,

5 $$ \alpha = \frac{\pi }{2} - \theta_{z} $$

Again, θ_z_ = $$\frac{\uppi }{2}$$ – α.

The value of this angle could be calculated directly from software using the latitude and longitude values.

### Solar azimuth angle

It is the solar angle on a horizontal plane, in degrees, between the line due south and the projection of sun's rays on the horizontal plane. It is denoted by r_s_^32^.

In other words, it is a horizontal angle measured from North to the horizontal projection of sun’s rays.

When measured from South towards West the solar azimuth angle is positive.

From the Fig. 13, Solar Azimuth Angle, r_s_ is ∠BAS.

AS is Line due on the South and BA is Horizontal projection line of Sun’s ray towards West.

Therefore, r_s_ is ∠BAS.

The value of this angle could also be calculated directly from software using the latitude and longitude values.

### Incident angle (θ OR θ_i_)

**Figure 14:**
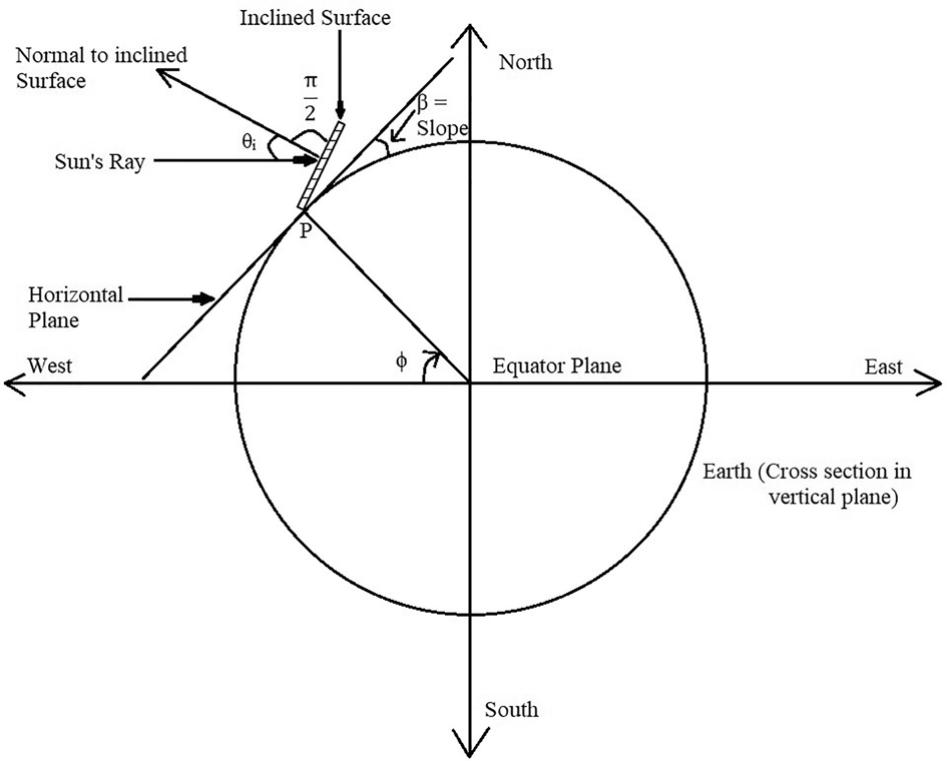
Angle of incidence (θ_i_), tilt angle (β) and angle of latitude (ϕ_L_)^32^.

It is the angle between the incident sun rays on the plane surface and the normal on that surface. It is denoted by θ_i_^32^. The angle of incidence is shown in Fig. 14.

In general, the angle of incidence can be expressed as

6 $$ {\text{cos }}\,\theta_{{\text{i}}} = {\text{ cos}}\,\delta\, {\text{cos}}\, \omega\, \left({{\text{cos}}\,\phi {\text{ cos }}\,\beta \, + {\text{ sin}}\,\phi {\text{ sin }}\,\beta {\text{ cos r}}} \right) \, + {\text{ cos}}\,\delta {\text{ sin}}\,\omega {\text{ sin }}\,\beta {\text{ sin r }} + {\text{ sin}}\,\delta ({\text{ sin}}\,\phi {\text{ cos }}\,\beta \, {-}{\text{  cos}}\,\phi {\text{ sin}}\,\beta {\text{ cos r}}) $$

### The slope (optimal tilt angle) (β)

It is the angle between inclined plane surface with the horizontal. It is denoted by β^32^.

It is taken to be positive for the surface slopping towards the south and negative for the surface slopping towards the south^32^.

β is the angle between inclined surface and the horizontal plane with respect to the collector surface.

## Calculation of solar angles required for the present business complex

**Figure 15:**
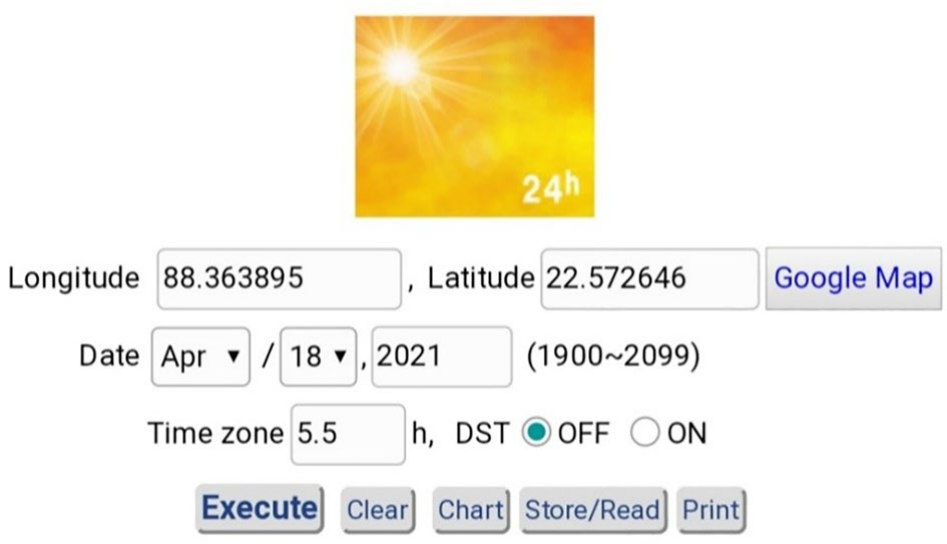
Given specifications of Kolkata^34^.

Calculation of the solar angles for the location of the business complex i.e., Kolkata (Longitude is 88.363895° E, Latitude is 22.572646° N) at a given time i.e., 12:00 noon on 18 April 2021 is done. To calculate solar elevation angle and azimuth angle the software Keisan Online Calculator will be used. Figure 15 shows the given specifications of Kolkata presented in software.

**Figure 16:**
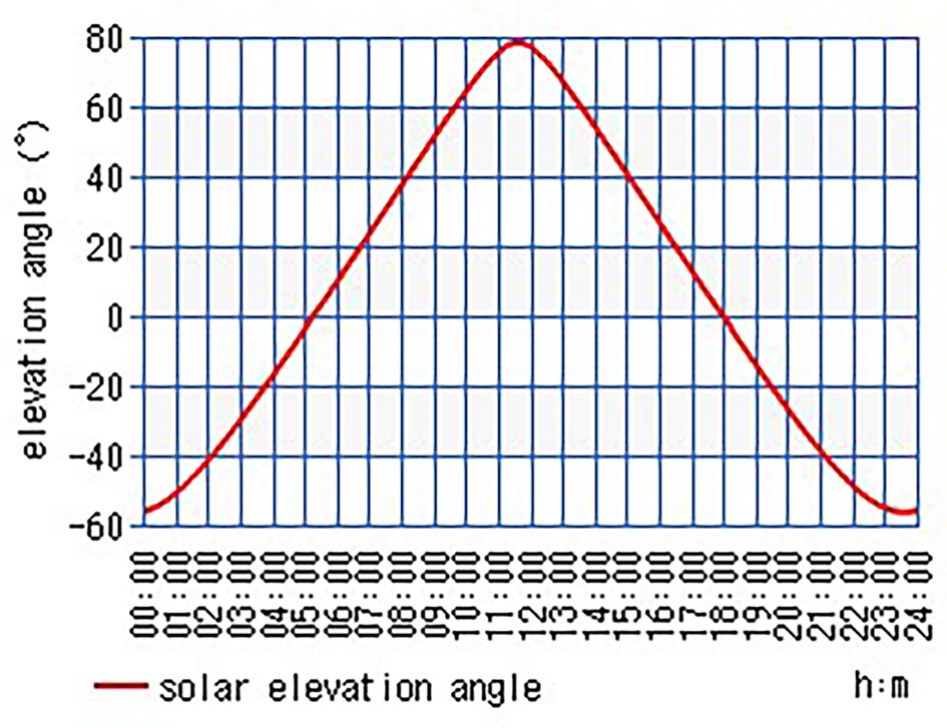
Graphical representation of solar elevation angles of Kolkata on 18 April 2021^34^.

Figure 16 shows the variation of solar elevation angle with respect to time at Kolkata on 18 April 2021. Here, X axis represents time and Y axis is the variation of solar elevation angle. It is observed from the graph that the value of elevation angle steadily begins to increase from negative at 00:00 h after that it becomes positive at around 05:00 h then reach its peak around 11:30 h and after that it steadily decreases to again become negative around 18:00 h. As the solar elevation angle represents how high sun appears in the sky. So, it could be concluded from the graph that the sun is below the horizon before 05:00 h and after 18:00 h and it reaches its highest at 11:30 h. Hence, the solar energy could be harvested between 05:00 h to 18:00 h. The values of the solar elevation angle (α) and azimuth angle (r_s_) of a given location, here, Kolkata and at a given day, here, 18 April 2021 can be directly calculated by the latitude and longitude values by the calculator. The calculated values of the Solar Angles are predicted in Table 3.

**Table 3:** Value of the solar angles.

Sl. no	Parameters	Symbol	Value
1	Air mass	m	1.0262628
2	Altitude angle or solar elevation angle	α	77.01°
3	Zenith angle	θ_z_	12.99°
4	Latitude angle	ϕ	22.572646°
5	Declination angle	δ	10.5334°
6	Incident angle	θ_i_	60.198°
7	Hour angle	ω	0°
8	Optimal tilt angle	β	49.3153814°
9	Azimuth angle	r_s_	27.30°

## Performance ratio and daily power output of the PV system

**Figure 17:**
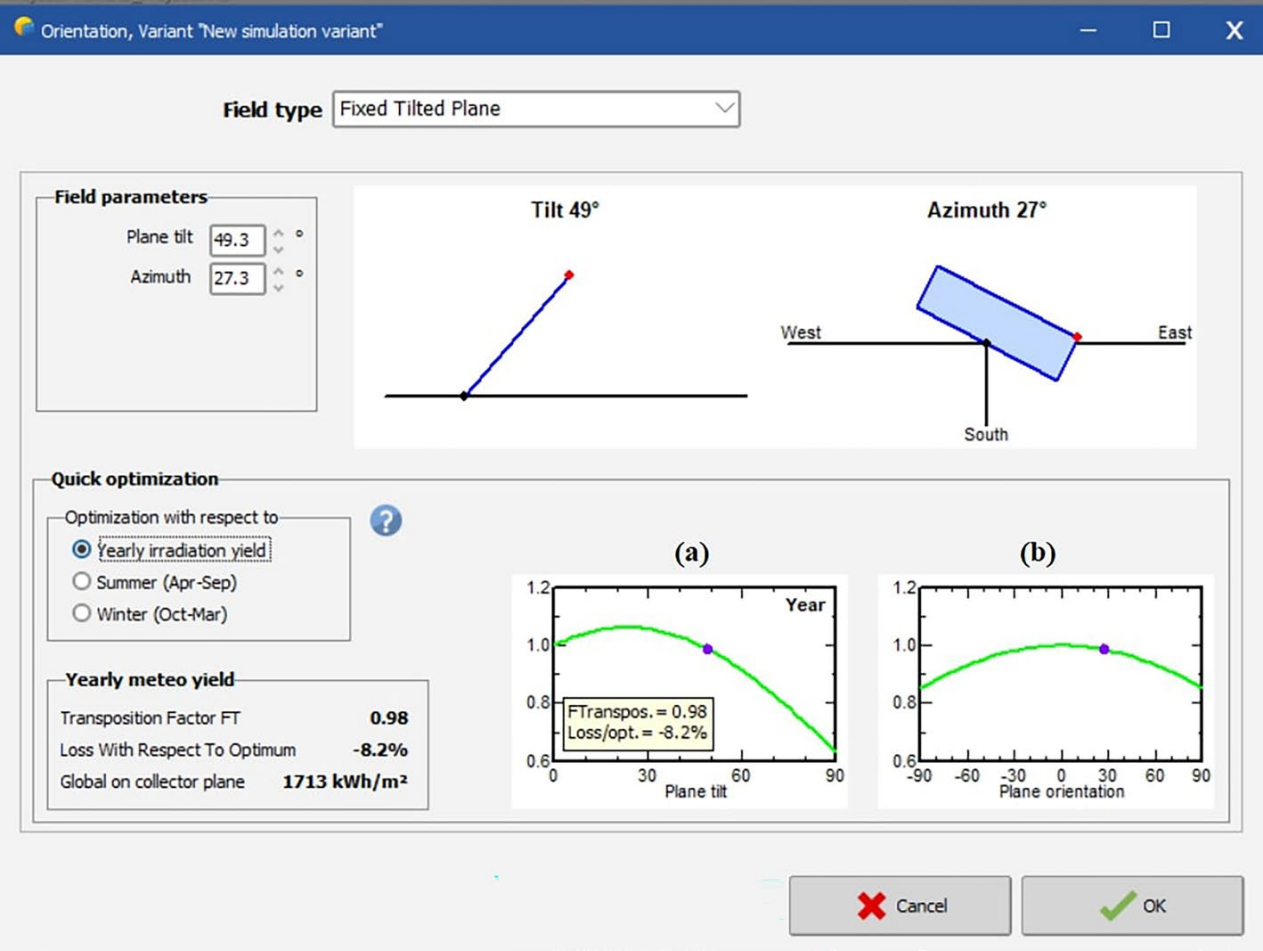
Defining Tilt angle and Azimuth angle in software.

Now, in order to determine the performance ratio of the PV system of the business complex the values of the solar angles that were calculated till now will be put in the software PVSyst. The city being considered is Kolkata, India, the location of the business complex. Some of the important screenshots of simulation are as follows:

Figure 17 shows the tilt angle and azimuth angle of the solar PV system. The plane considered here is a fixed tilted plane. The optimization is done with respect to the yearly irradiation yield. The X axis of the graph (a) represents the plane tilt whereas the X axis of the graph (b) represents the plane orientation and the Y axis of both the graphs represents the yearly irradiation yield. From the simulation the values obtained of transposition factor (TF) is 0.98, losses with respect to optimum is -8.2% and irradiation on collector plate is 1713 kWh/m^2^.

**Figure 18:**
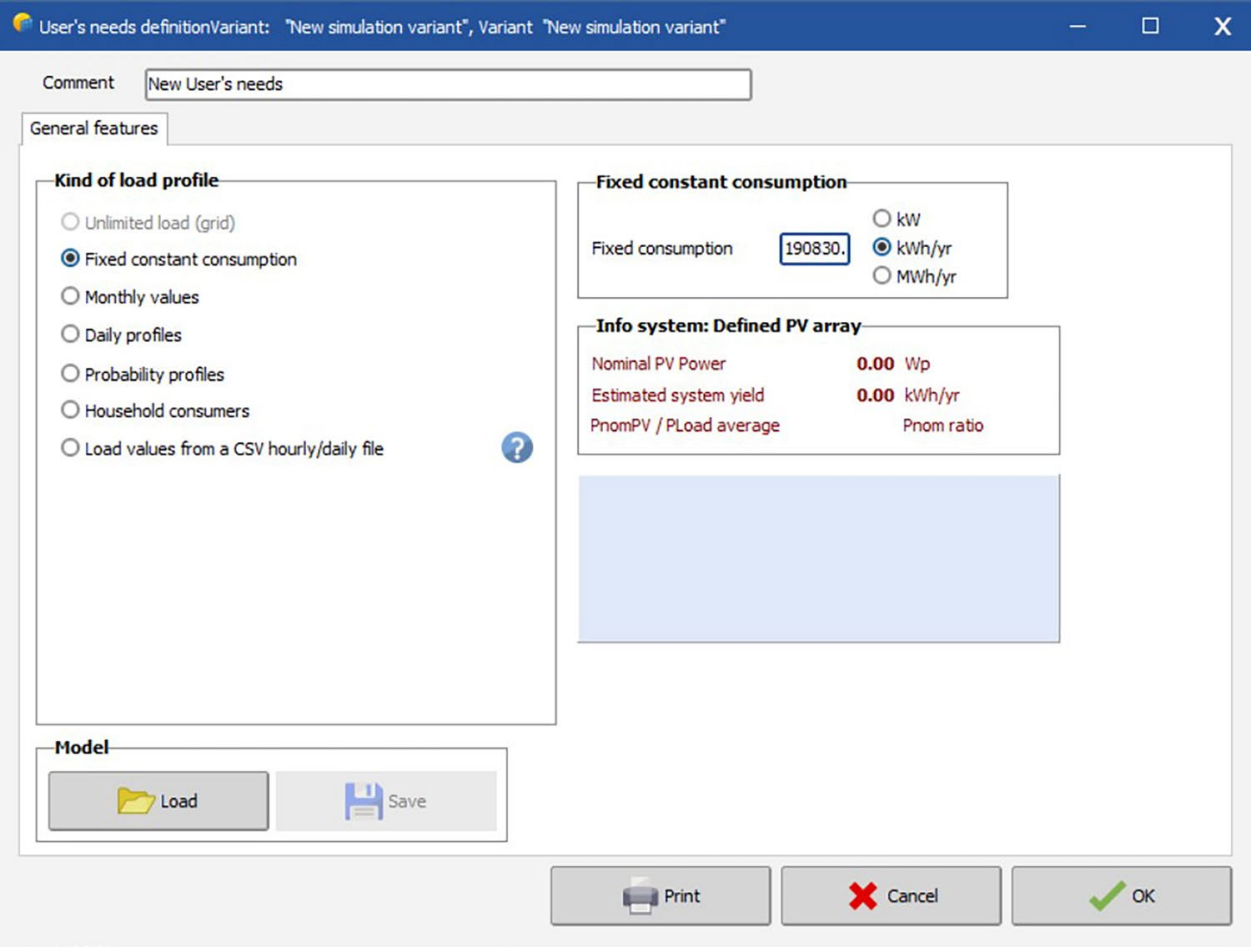
Defining total power consumption in software.

Figure 18 represents in the input of the total Load of the business complex in a year which is found out to be 1,90,830.760 Kilowatt-hours/year.

**Figure 19:**
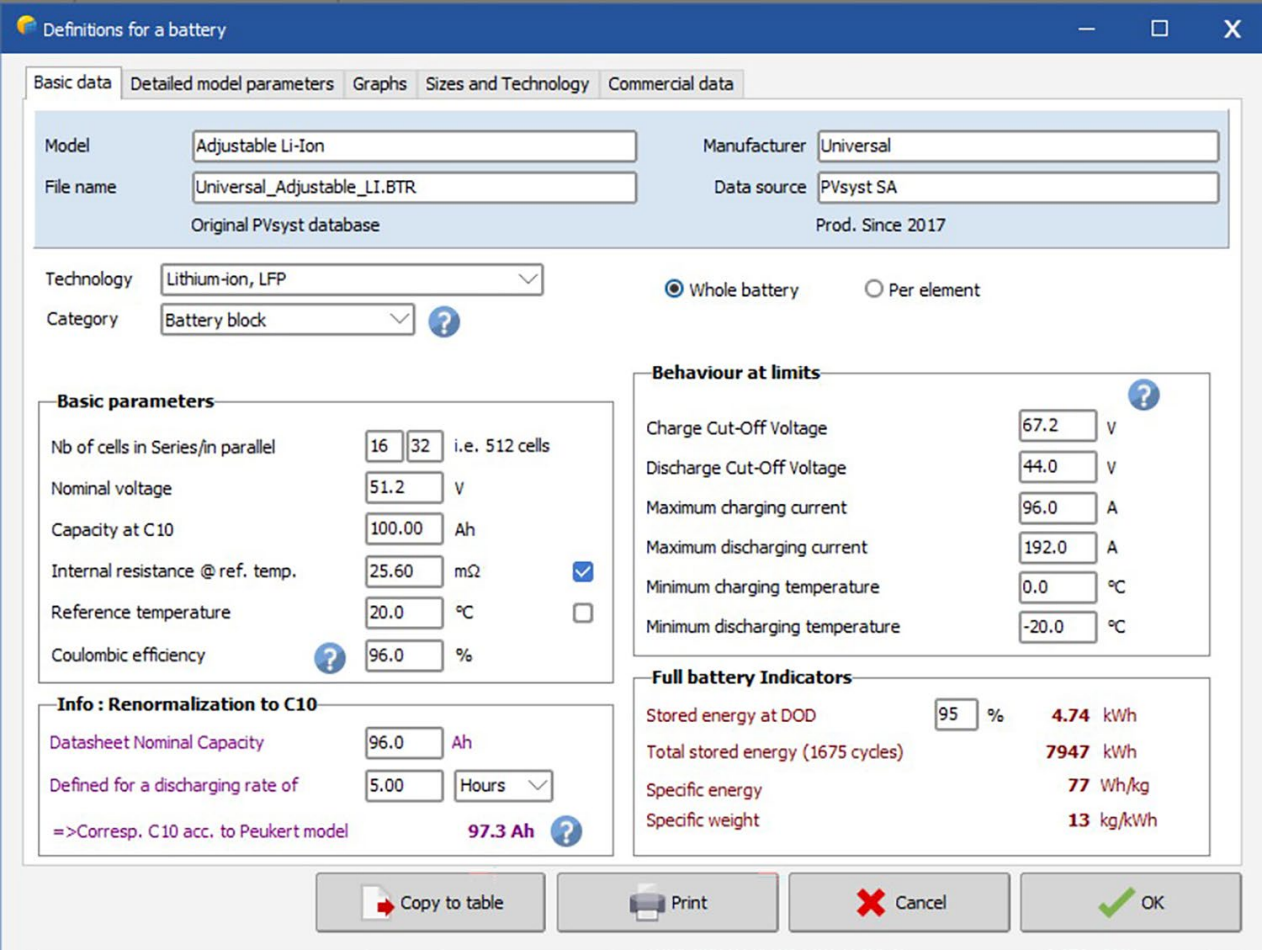
Defining battery requirement in software.

Figure 19 shows the battery specifications used in the solar PV plant. The battery used here is an adjustable lithium-ion battery. The total no. of cells used here is 512 of which 16 are connected in series and 32 are connected in parallel.

**Figure 20:**
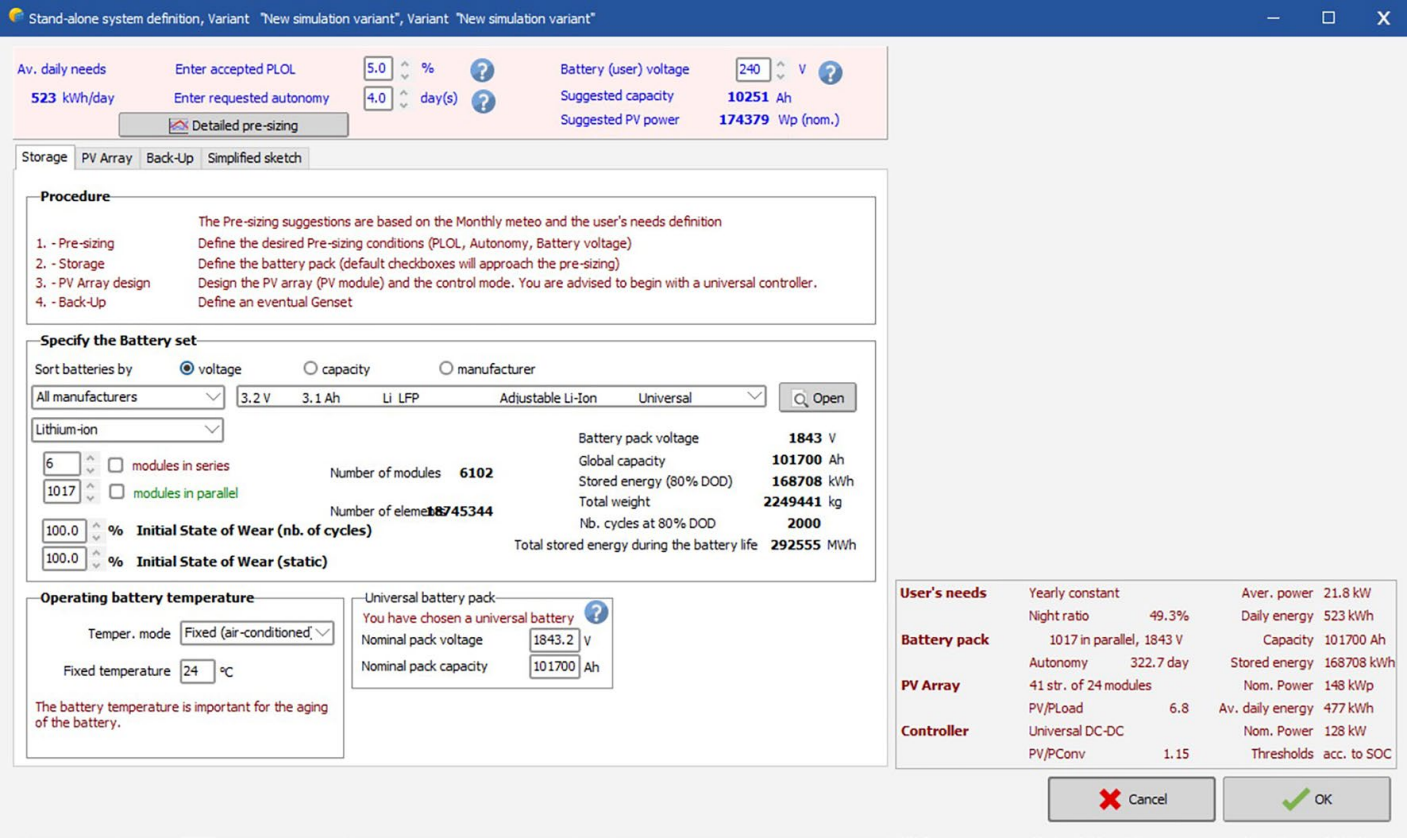
Defining number of modules requirement in software.

Figure 20 defines the number of solar modules used here. As calculated previously the required number of solar modules to meet the complete energy demand of the business complex is 6097. But here 6102 modules have been considered in order to maintain the series and parallel symmetry. But it would also be beneficial as it will serve as a backup whenever there is some extra energy demand. Among the 6102 modules 6 modules will be connected in series row and 1017 modules would be connected in parallel row. It also shows the battery operation specifications used in the solar PV plant. The battery is to be operated at standard room temperature at 24 °C in a fixed air-conditioned room. It is because battery temperature is crucial for the aging of the battery.

**Figure 21:**
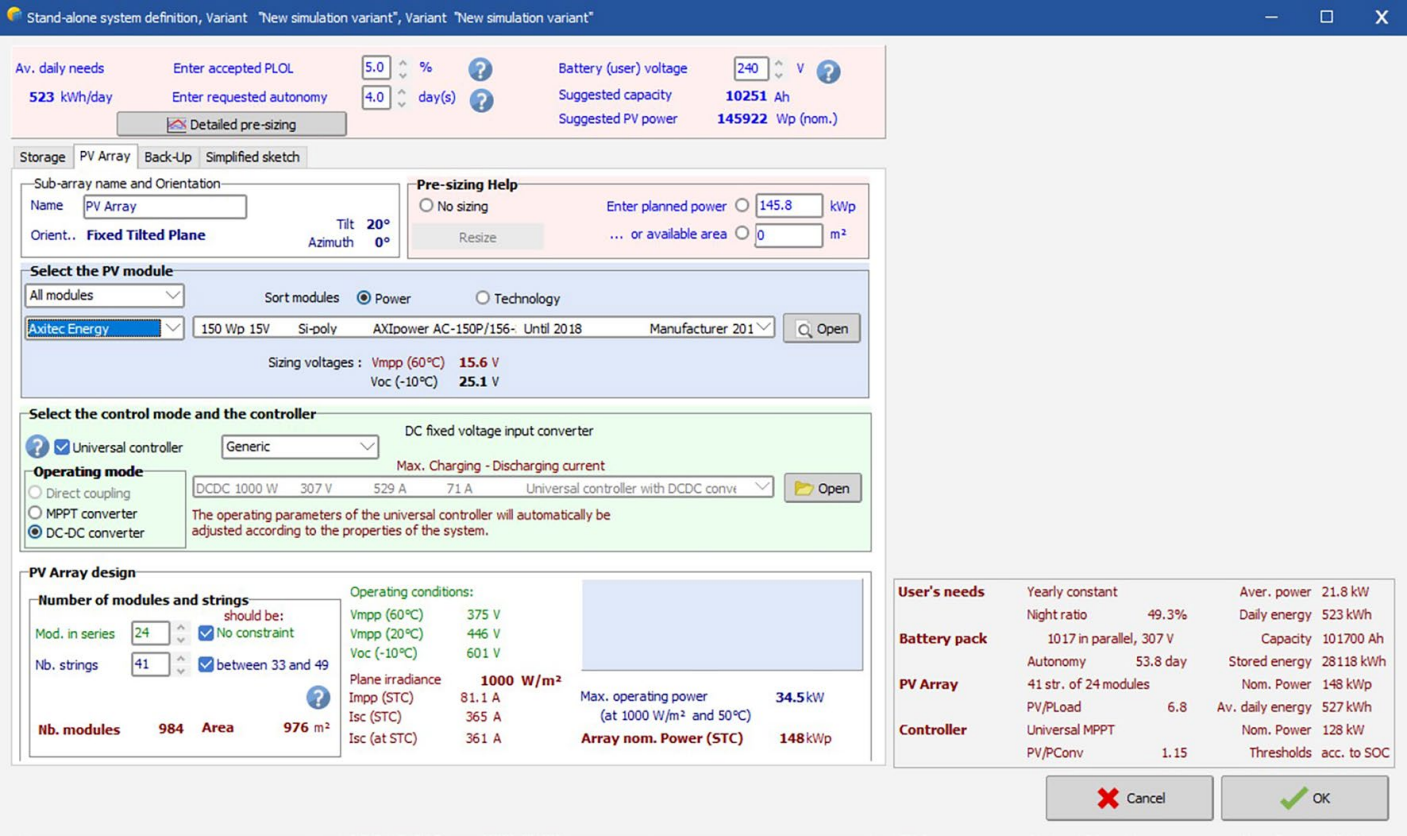
Defining PV cell requirement in software.

Figure 21 defines the specifications of PV cell used. As defined is the calculation the PV cell used here is a 150 W polycrystalline silicon cell. The PV array design is also given here. The array would have 41 stings and 24 modules in series. It is done so that there is optimum used of space i.e., minimum area used for maximum electricity generation. The figure also shows that the controller used in the system is a universal controller which will operate in DC-DC converter mode.

**Figure 22:**
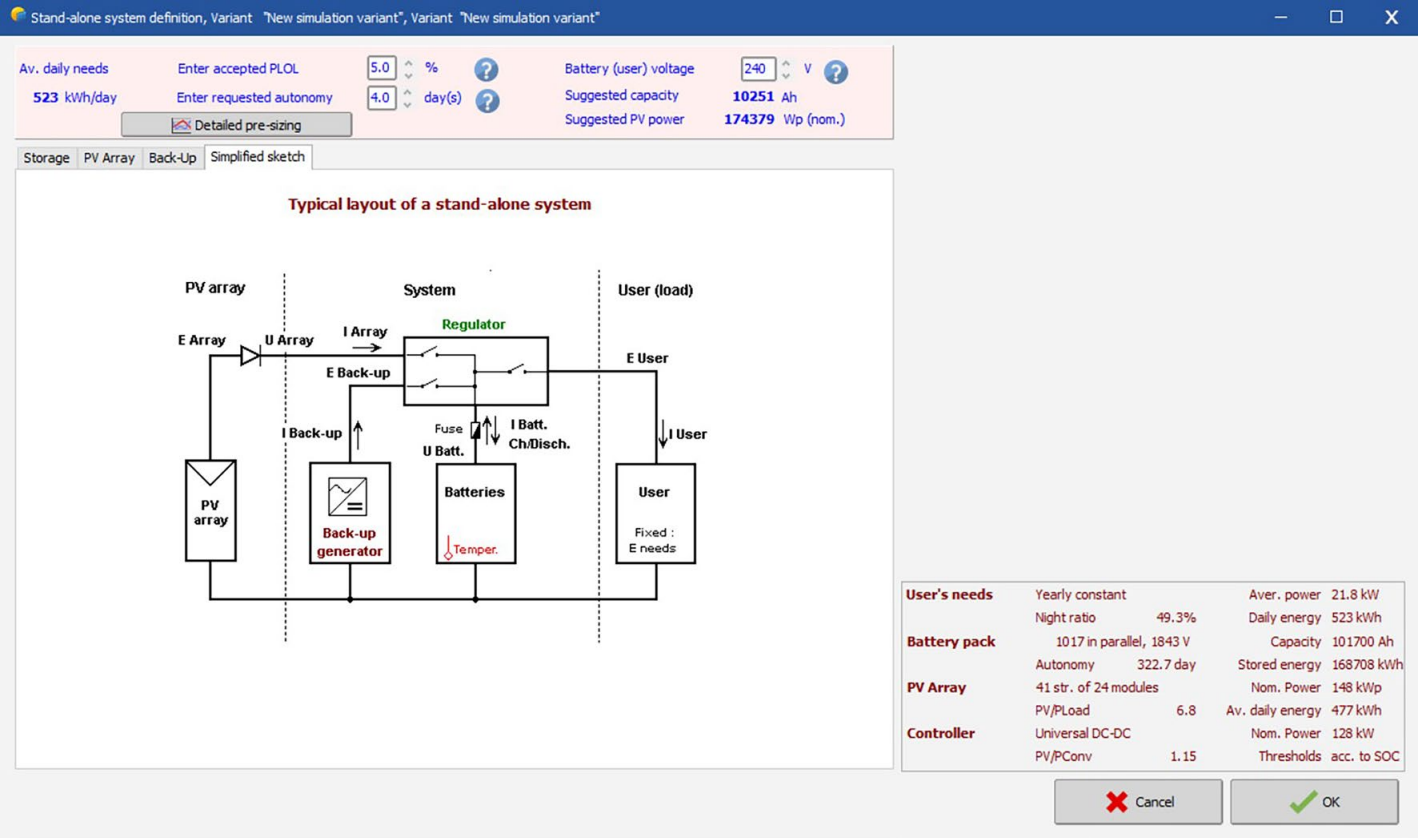
Circuit diagram of the PV system.

Figure 22 shows the circuit diagram of the stand-alone solar PV system for the proposed business complex.

**Figure 23:**
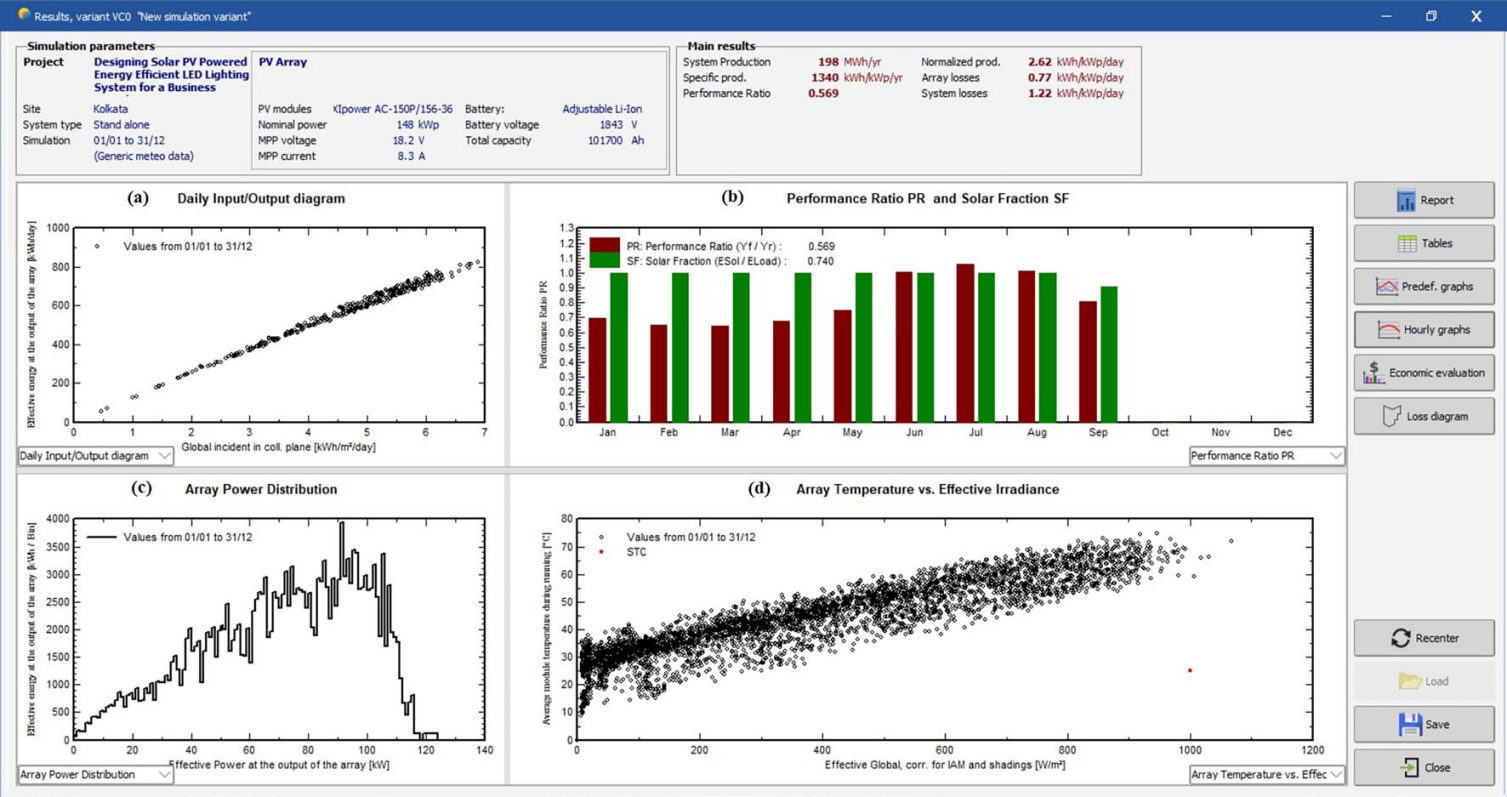
Software generated report for stand-alone PV system.

Figure 23 shows the final software generated results for the stand-alone PV system.

Considering first graph, 23(a), the daily input/output diagram, the X axis represents the global incident on collector plate in kWh/m^2^/day and the Y axis represents the effective energy at the output of the array in kWh/day. It is observed that the output energy increases with the increase in incident solar radiation.

The graph 23(b) is the performance ratio (PF) and solar fraction (SF) graph. Here, the X axis represents the performance ratio (PF) and the Y axis represents the time in months. From the simulation, the PF value is obtained as 0.569 and the solar fraction (SF) is 0.740.

The graph 23(c) is the array of power distribution. The X axis represents the effective power at the output of the array in kW and the Y axis represents the effective energy at the output of the array in kWh. The present curve drawn here is for the entire year, from 1st January to 30th December, 2021.

The graph 23(d) is the array temperature versus effective irradiance. The X axis represents the effective irradiance in W/m^2^ of the array in kW and the Y axis represents the average module temperature during running in °C. It is estimated for the entire year from 1st January to 30th December, 2021. From the graph, it is established that the temperature of the module increases with increase in solar irradiation but the performance decreases. The parameters obtained from the simulation are shown in Table 4.

**Table 4:** From the simulation the parameters obtained.

Sl. no	Parameters	Value
1	Transposition factor (TF)	0.98
2	Solar fraction (SF)	0.740
3	Performance ratio (PR)	0.569

## Cost estimation

Each energy-producing organization has their own cost per unit of electricity. However, here we will determine the unit rate without considering debits, capital and taxes. On considering our stand-alone solar PV plant for the business complex, the breakup of the capital cost is shown in Table 5.

**Table 5:** Cost estimation of the proposed complex.

Sl. no	Parameters	Value
1	Total load of the business complex	522.824 Kilowatt-hours/day
2	Safety factor (for safety we will be calculating the prices for double the load)	1 MW
3	Cost of a microtek 150 W/12 V polycrystalline panel	₹ 8,000
4	Total cost of solar panel	₹ 4,88,16,000
5	Cost of inverter (500 kW)	₹ 33,00,000
6	Cost of mounting structure	₹ 31,36,944
7	Miscellaneous costs (cables and distribution boxes)	₹ 2,87,55,320
8	Cost of installation	₹ 4,44,40,040
9	The total cost	₹ 12,84,08,304

## Conclusion

Based on the findings of this paper, the feasibility of designing a stand-alone solar photovoltaic (PV) system is evaluated which can meet the entire energy requirement of a proposed business complex. It has been carried out without the support of any conventional supply of energy, i.e., conventional power plant.

The paper starts with a brief theory of solar photovoltaic power plant, its working principle, application and percentage share in energy sector. Then descriptions of different solar plants along with their working principle is presented in a brief but compact manner. Construction of a solar plant is also specified in the study as they play an important role. In the second phase, a typical layout of a business complex is shown and the annual energy demand of the business complex is calculated. Specific attention was paid to the usage of LED lights for illumination purposes as they play an important role in energy conservation. It also contains the solar radiation map of India. The crucial factors including designing the PV system is that the theory and calculation of the solar angles. The properties of the various solar angles are described for certain important applications. The calculated value of Optimal Tilt Angle is 49.3153814° considering the business complex in Kolkata (India). The simulation technique and practicality of the system is also described in a step by step process. The paper concludes with a cost estimation of the solar PV system for a particular building.

The main conclusions from this study are as follows:

i. The stand-alone solar photovoltaic (PV) system would be able to supply complete energy independence for any individual building. Hence, the non-conventional energy (here solar energy) may be the alternative source of illumination and utilities for an office complex or a residential building.
ii. The value of Transposition Factor (0.98) establishes the efficiency of the PV system, the Solar Fraction (0.740) value establishes the effectiveness of the system and the Performance Ratio (0.569) shows that energy independence is a possibility in the future. Furthermore, it could also be emphasized that as the efficiency of the PV cells increases so it would be the value of Performance Ratio and Solar Fraction of the system.
iii. The cost estimation shows that the acquisition and installation cost of PV system is economically feasible for a typical business complex.

The maintenance needed for solar power plants is very less and therefore, it can be installed at any given space where sufficient sun light is available. It can be easily installed on the rooftop of high-rise buildings. Hence, it is concluded that solar energy generated through solar PV modules is the energy of the future. This system will reduce pollution significantly. It is very much possible to use them in individual building to attain complete energy independence in the future.

## Supplementary Information

Supplementary Information.

## Data Availability

All data generated or analysed during this study are included in this published article [and its supplementary information files].
