# Supplementary material for: Design of LED lighting system using solar powered PV cells for a proposed business complex
Source: Sci Rep. 2022 Aug 2;12:13289. doi: 10.1038/s41598-022-17353-2 (PMC9345941; doi:10.1038/s41598-022-17353-2)
Supplement: Supplementary file 1 — Supplementary Information. [file 41598_2022_17353_MOESM1_ESM.docx]

1. **Total Power Calculation of** **The Business Complex –**

Table S1 – Appliances used in the Business Complex with their Power Rating [1, 2]

| **Sl. No.** | **Appliance** | **Quantity** | **Individual Power Rating (Watt)** |
| --- | --- | --- | --- |
| 1 | LED Bulb – 40 Watt Equivalent | 21 | 10 |
| 2 | LED Bulb – 60 Watt Equivalent | 132 | 13 |
| 3 | LED Bulb – 75 Watt equivalent | 29 | 18 |
| 4 | LED Bulb – 100 Watt Equivalent | 3 | 23 |
| 5 | Blender | 3 | 500 |
| 6 | Coffee Machine | 2 | 1000 |
| 7 | Freezer – Chest – 15 cu. ft. | 2 | 1080 |
| 8 | Garbage Disposal | 1 | 450 |
| 9 | Kettle – Electric | 1 | 1200 |
| 10 | Microwave | 2 | 1000 |
| 11 | TV – LCD | 2 | 150 |
| 12 | Cable Box | 2 | 35 |
| 13 | Central Air Conditioner – 24,000 BTU NA | 2 | 3800 |
| 14 | Vacuum | 4 | 1000 |
| 15 | Desktop Computer (Standard) | 13 | 200 |
| 16 | Printer | 13 | 100 |
| 17 | Elevator | 1 | 7631 (in kWh) |
| 18 | Box Fan | 1 | 200 |
| 19 | Furnace Fan Blower | 8 | 800 |
| 20 | Smart Phone – Recharge | 26 | 6 |

Total Load of an Appliance = Number of appliances × Load of the appliance × No. of hours used

Total Load of the Business Complex =

21×10×14 + 132×13×14 + 29×18×14 + 3×23×6 + 3×500×10 + 2×1000×10 + 2×1080×24 + 1×450×2 + 1×1200×4 + 2×1000×10 + 2×150×14 + 2×35×14 + 2×3800×14 + 4×1000×2 + 13×200×14 + 13×100×14 + 1×7631×14 + 1×200×14 + 8×800×14 + 26×6×14

= 5,22,824 Watt-hours

Total Load of the Business Complex in a day = 5,22,824 Watt-hours/day

= 522.824 Kilowatt-hours/day

Total Load of the Business Complex in a year = 5,22,824 Watt-hours × 365

= 19,08,30,760 Watt-hours/year

= 1,90,830.760 Kilowatt-hours/year

= 190.831 Megawatt-hours/year

1. **Calculation of PV Cells** **Required for The Business Complex –**

|  | 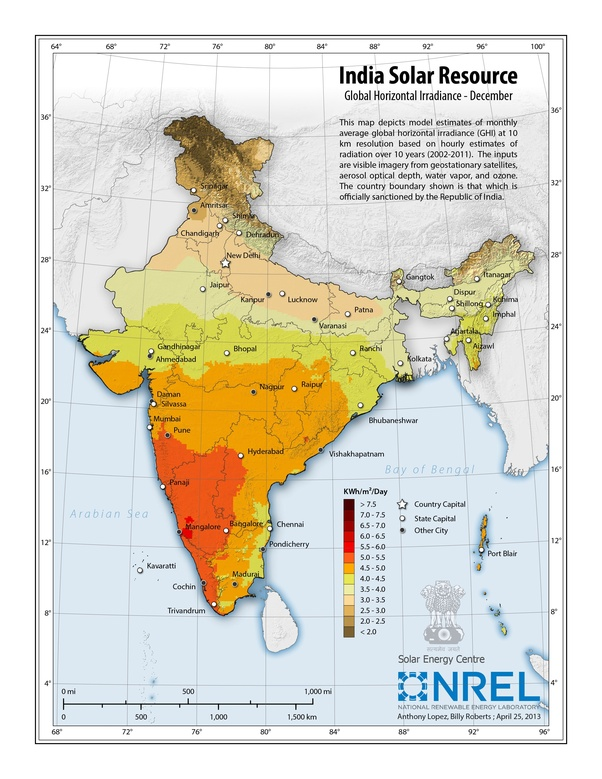 |
| --- | --- |

Figure S1 - Solar radiation map of India [3]

Solar Irradiance in Kolkata = 3.5 – 4.0 kWh/m^2^/day

Annual full sun hours in Kolkata = 3.5 × 365 = 1227.5 kWh/m^2^/year [From Figure S1]

Efficiency of PV cell considering it a polycrystalline cell = 17% [4]

PV System Size = $\frac{\frac{19,08,30.760}{1227.5}}{0.17}$ = 914.4878 kW

**No of modules =** $\frac{914487.8}{150}$ = 6096.5853 ≃ 6097 [Module power = 150W [5]]

Therefore, we would require 6097 panels to supply complete power to the business complex.

1. **Calculation of Solar Angles Required for The Business Complex –**

Calculation of the solar angles for the location of the business complex i.e., Kolkata (Longitude – 88.363895° E, Latitude – 22.572646° N) at a given time i.e., 12:00 p.m. noon on 18 April 2021 will be done. To calculate solar elevation angle and azimuth angle the software Keisan Online Calculator will be used.


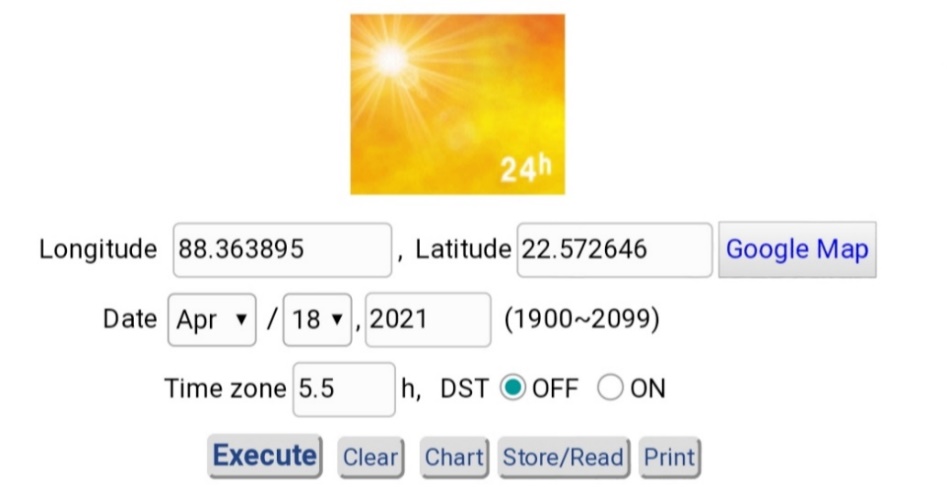


Figure S2 – Given Specifications of Kolkata [4]


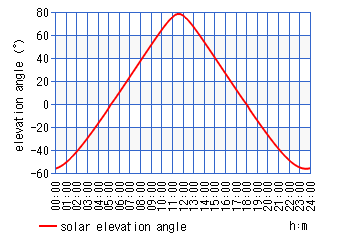


Figure S3 – Graphical Representation of solar elevation angles of Kolkata on 18 April 2021 [6]

Figure S3 shows the variation of solar elevation angle with respect to time at Kolkata on 18 April 2021. Here, –X axis represents time and –Y axis is the variation of solar elevation angle. It could be observed from the graph that the value of elevation angle steadily begins to increase from negative at 00:00 hours after that it becomes positive at around 05:00 hours then reaches its peak around 11:30 hours and after that it steadily decreases to again become negative around 18:00 hours. As the solar elevation angle represents how high sun appears in the sky. So, it could be concluded from the graph that the sun is below the horizon before 05:00 hours and after 18:00 hours and it reaches its highest at 11:30 hours. Hence, the solar energy could be harvested between 05:00 hours to 18:00 hours.

The values of the solar elevation angle (α) and azimuth angle (r_s_) of a given location, here, Kolkata and at a given day, here, 18 April 2021 can be directly calculated using the latitude and longitude values using the calculator. The values of the two angles with respect to the time of that particular day is given in Table S2.

Table S2 – Tabular Representation of solar elevation angles of Kolkata on 18 April 2021 [6]

| **hh:mm** | **Elevation angle (α)** | **Azimuth angle (r_s_)** |
| --- | --- | --- |
| 00:00 | -56.18 | 10.63 |
| 00:15 | -55.34 | 17.01 |
| 00:30 | -54.15 | 23.06 |
| 00:45 | -52.64 | 28.69 |
| 01:00 | -50.84 | 33.88 |
| 01:15 | -48.78 | 38.62 |
| 01:30 | -46.52 | 42.94 |
| 01:45 | -44.07 | 46.85 |
| 02:00 | -41.47 | 50.40 |
| 02:15 | -38.74 | 53.64 |
| 02:30 | -35.90 | 56.58 |
| 02:45 | -32.97 | 59.28 |
| 03:00 | -29.95 | 61.76 |
| 03:15 | -26.87 | 64.05 |
| 03:30 | -23.73 | 66.17 |
| 03:45 | -20.54 | 68.15 |
| 04:00 | -17.31 | 70.01 |
| 04:15 | -14.05 | 71.77 |
| 04:30 | -10.76 | 73.43 |
| 04:45 | -7.44 | 75.02 |
| 05:00 | -3.94 | 76.54 |
| 05:15 | -0.03 | 78.01 |
| 05:30 | 3.03 | 79.43 |
| 05:45 | 6.34 | 80.82 |
| 06:00 | 9.72 | 82.19 |
| 06:15 | 13.13 | 83.54 |
| 06:30 | 16.57 | 84.88 |
| 06:45 | 20.01 | 86.23 |
| 07:00 | 23.46 | 87.59 |
| 07:15 | 26.92 | 88.98 |
| 07:30 | 30.38 | 90.40 |
| 07:45 | 33.84 | 91.88 |
| 08:00 | 37.30 | 93.43 |
| 08:15 | 40.75 | 95.08 |
| 08:30 | 44.19 | 96.84 |
| 08:45 | 47.62 | 98.76 |
| 09:00 | 51.04 | 100.89 |
| 09:15 | 54.42 | 103.27 |
| 09:30 | 57.77 | 106.01 |
| 09:45 | 61.08 | 109.22 |
| 10:00 | 64.31 | 113.07 |
| 10:15 | 67.44 | 117.82 |
| 10:30 | 70.41 | 123.86 |
| 10:45 | 73.16 | 131.75 |
| 11:00 | 75.53 | 142.23 |
| 11:15 | 77.32 | 155.99 |
| 11:30 | 78.26 | 172.89 |
| 11:45 | 78.15 | 190.97 |
| 12:00 | 77.01 | 207.30 |
| 12:15 | 75.07 | 220.34 |
| 12:30 | 72.61 | 230.21 |
| 12:45 | 69.81 | 237.65 |
| 13:00 | 66.79 | 243.39 |
| 13:15 | 63.64 | 247.93 |
| 13:30 | 60.39 | 251.63 |
| 13:45 | 57.08 | 254.72 |
| 14:00 | 53.72 | 257.38 |
| 14:15 | 50.33 | 259.71 |
| 14:30 | 46.91 | 261.78 |
| 14:45 | 43.48 | 263.67 |
| 15:00 | 40.04 | 265.41 |
| 15:15 | 36.59 | 267.03 |
| 15:30 | 33.13 | 268.57 |
| 15:45 | 29.67 | 270.04 |
| 16:00 | 26.22 | 271.46 |
| 16:15 | 22.76 | 272.84 |
| 16:30 | 19.31 | 274.21 |
| 16:45 | 15.88 | 275.56 |
| 17:00 | 12.45 | 276.91 |
| 17:15 | 9.04 | 278.26 |
| 17:30 | 5.67 | 279.64 |
| 17:45 | 2.40 | 281.04 |
| 18:00 | -0.62 | 282.48 |
| 18:15 | -4.66 | 283.96 |
| 18:30 | -8.08 | 285.50 |
| 18:45 | -11.38 | 287.11 |
| 19:00 | -14.66 | 288.80 |
| 19:15 | -17.91 | 290.58 |
| 19:30 | -21.12 | 292.48 |
| 19:45 | -24.29 | 294.50 |
| 20:00 | -27.41 | 296.67 |
| 20:15 | -30.47 | 299.00 |
| 20:30 | -33.45 | 301.53 |
| 20:45 | -36.36 | 304.29 |
| 21:00 | -39.16 | 307.30 |
| 21:15 | -41.85 | 310.60 |
| 21:30 | -44.40 | 314.24 |
| 21:45 | -46.80 | 318.23 |
| 22:00 | -49.00 | 322.63 |
| 22:15 | -50.98 | 327.45 |
| 22:30 | -52.71 | 332.72 |
| 22:45 | -54.14 | 338.41 |
| 23:00 | -55.23 | 344.49 |
| 23:15 | -55.97 | 350.87 |
| 23:30 | -56.32 | 357.45 |
| 23:45 | -56.27 | 4.08 |
| 24:00 | -55.82 | 10.62 |

Here, Air Mass = m

Altitude angle or Solar Elevation Angle = α

Zenith Angle = θ_z_

Latitude Angle = ϕ

Declination Angle = 𝛿

Incident Angle = θ_i_

Hour Angle = ω

Optimal Tilt Angle = β

Azimuth angle = r_s_

From the table we got, α = 77.01°

θ_z_ = 90° - α

- - - - θ_z_ = 90° - 77.01°
      - θ_z_ = 12.99°

m = sec θ_z_

- - - - m = sec 12.99°
      - m = 1.0262628

From the table we got, r_s_ = 207.30°

- r_s_ = 207.30° - 180°
- r_s_ = 27.30°

Now, 𝛿 = 23.45 × sin [$\frac{360}{365}$(284 + n)]

- 𝛿 = 23.45 × sin [$\frac{360}{365}$(284 + 108)]
- 𝛿 = 10.5334°

At 12:00 p.m., ω = 0°

In summer, β = (22.572646°×0.9) + 29° = 49.3153814°

ϕ_L_ = 22.572646°

cos θ_i_ = cos 𝛿 cos ω (cos ϕ cos β + sin ϕ sin β cos r) + cos 𝛿 sin ω sin β sin r + sin 𝛿

(sin ϕ cos β – cos ϕ sin β cos r)

θ_i_ = cos^-1^ [cos 10.5334° cos 0° (cos 22.572646° cos 49.3153814° + sin 22.572646°

sin 49.3153814° cos 207.30°) + cos 10.5334° sin 0° sin 49.3153814° sin 207.30° + sin 10.5334° (sin 22.572646° cos 49.3153814° – cos 22.572646° sin 49.3153814° cos 207.30°)]

- θ_i_ = cos^-1^ [0.3375113369 + 0 + 0.159492994]
- θ_i_ = cos^-1^ [0.4970043309]
- θ_i_ = 60.198°

1. **Performance Ratio and Daily Power Output of the PV System –**

Now, to determine the performance ratio of the PV system of the business complex the values of the solar angles that were calculated till now will be put in the software PVSyst. The city being considered is Kolkata, India, the location of the business complex. Some of the important screenshots of simulation are as follows:


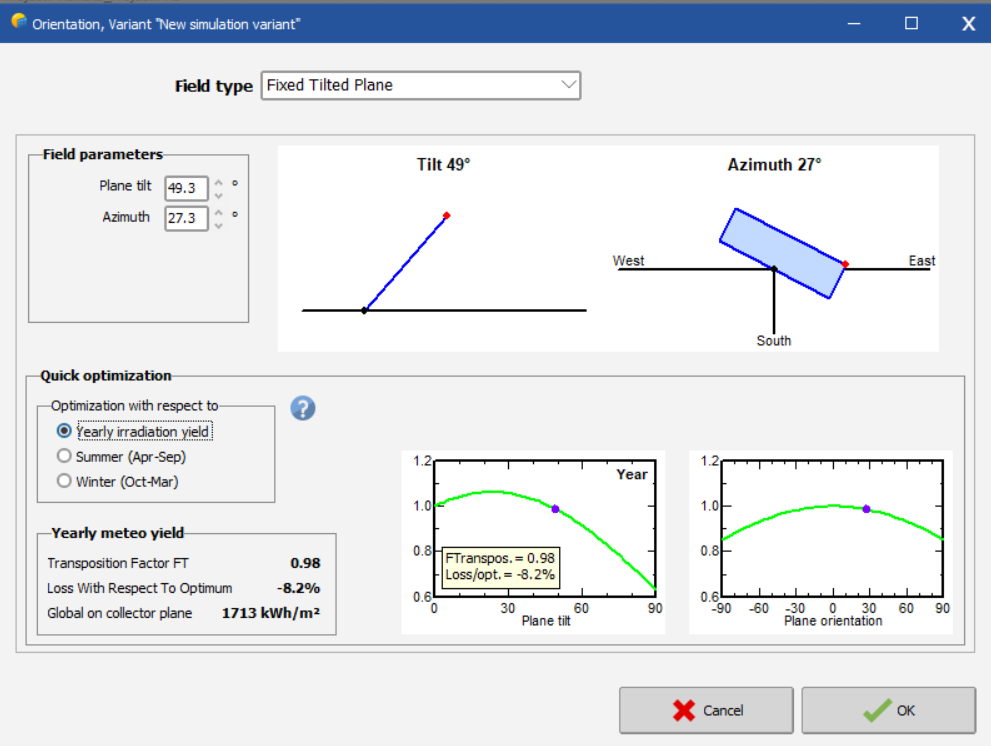


Figure S4 – Defining Tilt angle and Azimuth angle in software

Figure S4 shows the tilt angle and azimuth angle of the solar PV system. The plane considered here is a fixed tilted plane. The optimization is done with respect to the yearly irradiation yield. The –X axis of the left-hand side graph represents the plane tilt whereas the –X axis of the right-hand side graph represents the plane orientation and the –Y axis of both the graphs represents the yearly irradiation yield. From the simulation the values obtained of transposition factor (TF) is 0.98, losses with respect to optimum is -8.2% and irradiation on collector plate is 1713 kWh/m^2^.


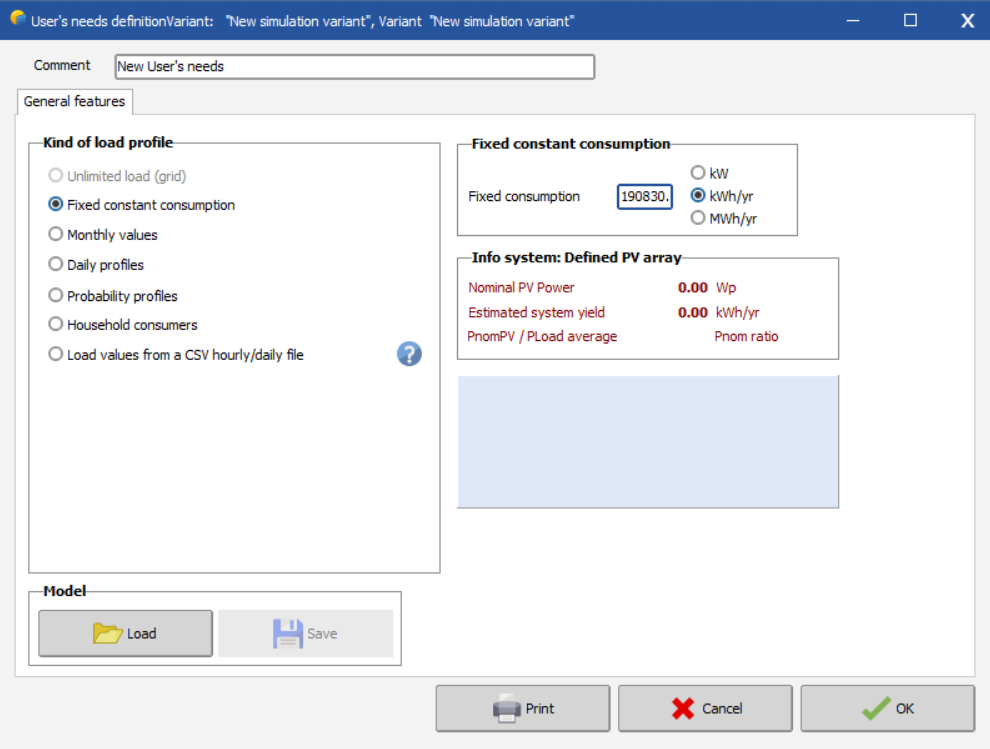


Figure S5 – Defining Total Power consumption in software

Figure S5 represents in the input of the total Load of the business complex in a year which is found out to be 1,90,830.760 Kilowatt-hours/year.


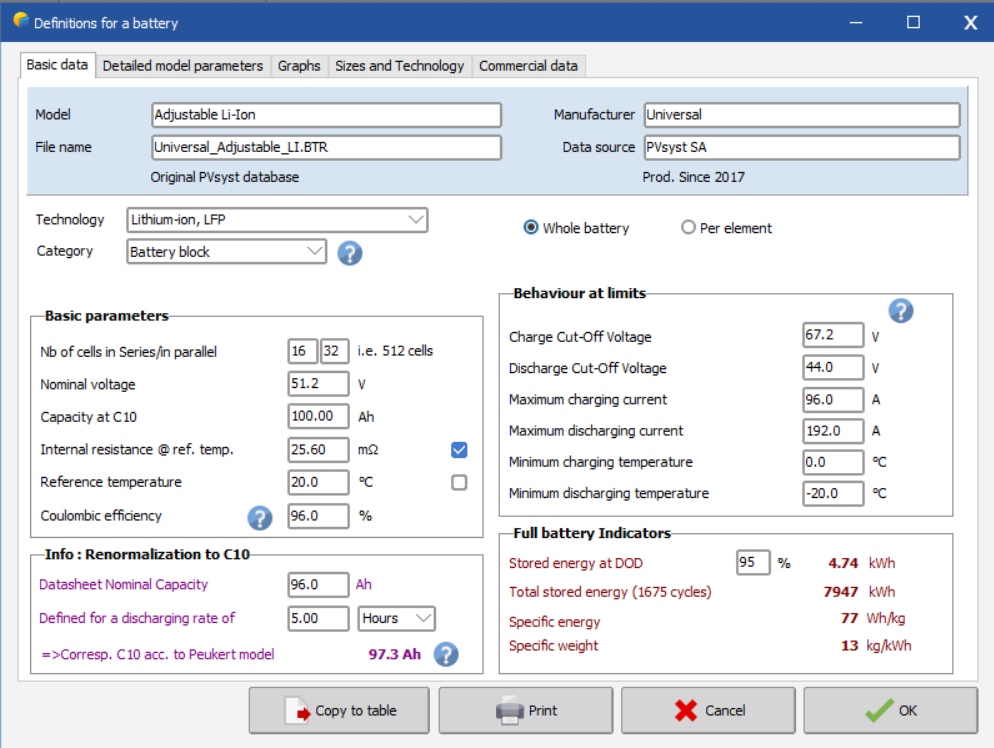


Figure S6 – Defining Battery requirement in software

Figure S6 shows the battery specifications used in the solar PV plant. The battery used here is an adjustable lithium-ion battery. The total no. of cells used here is 512 of which 16 are connected in series and 32 are connected in parallel.


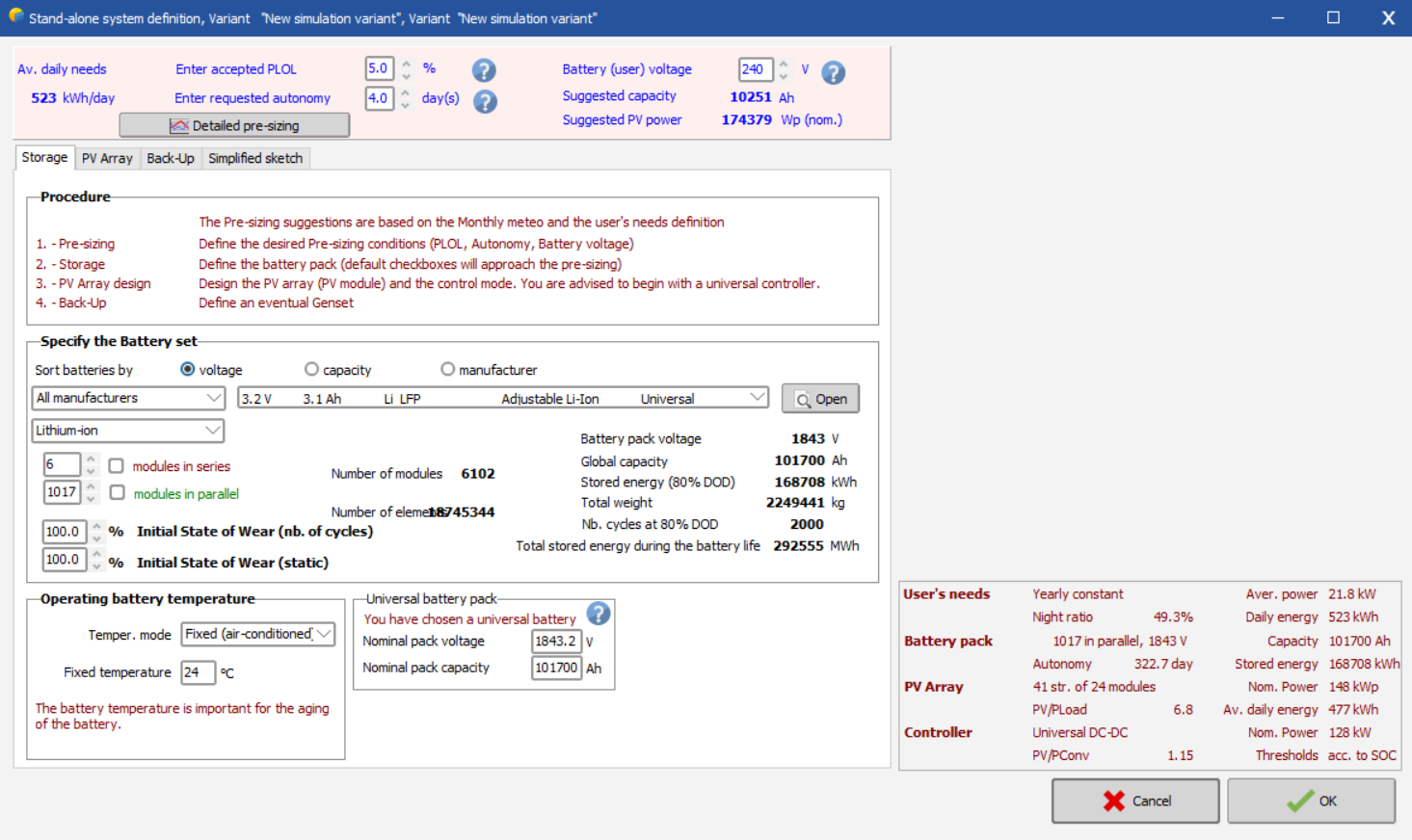


Figure S7 – Defining no. of Modules requirement in software

Figure S7 defines the number of solar modules used here. As calculated previously the required number of solar modules to meet the complete energy demand of the business complex are 6097. But here 6102 modules have been considered to maintain the series and parallel symmetry. But it would also be beneficial as it will serve as a backup whenever there is some extra energy demand. Among the 6102 modules 6 modules will be connected in series row and 1017 modules would be connected in parallel row. It also shows the battery operation specifications used in the solar PV plant. The battery is to be operated at standard room temperature at 24°C in a fixed air-conditioned room. It is because battery temperature is crucial for the aging of the battery.


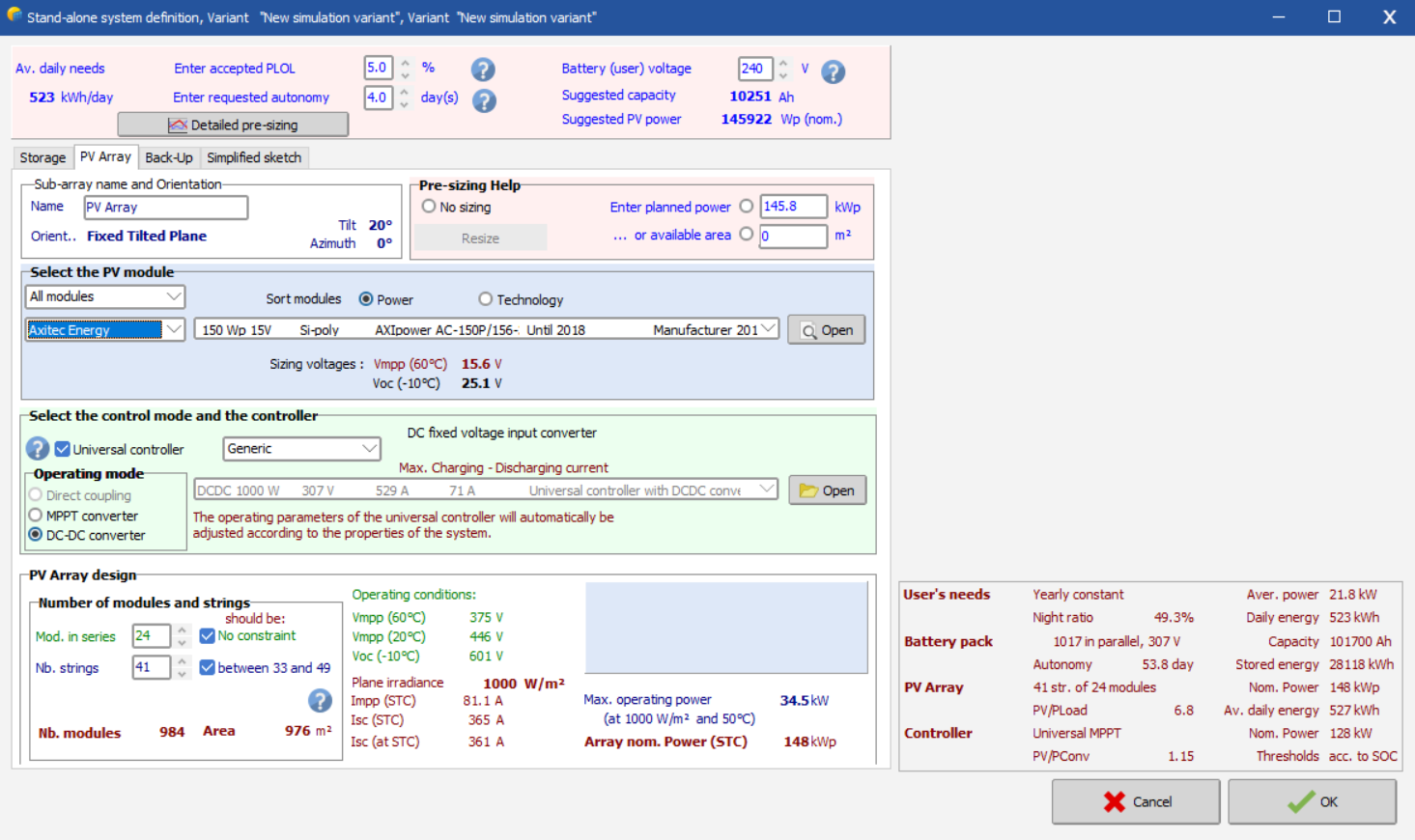


Figure S8 – Defining PV Cell requirement in software

Figure S8 defines the specifications of PV cell used. As defined is the calculation the PV cell used here is a 150 W poly crystalline silicon cell. The PV array design is also given here. The array would have 41 stings and 24 modules in series. It is done so that there is optimum used of space i.e., minimum area used for maximum electricity generation. The figure also shows that the controller used in the system is a universal controller which will operate in DC-DC converter mode.


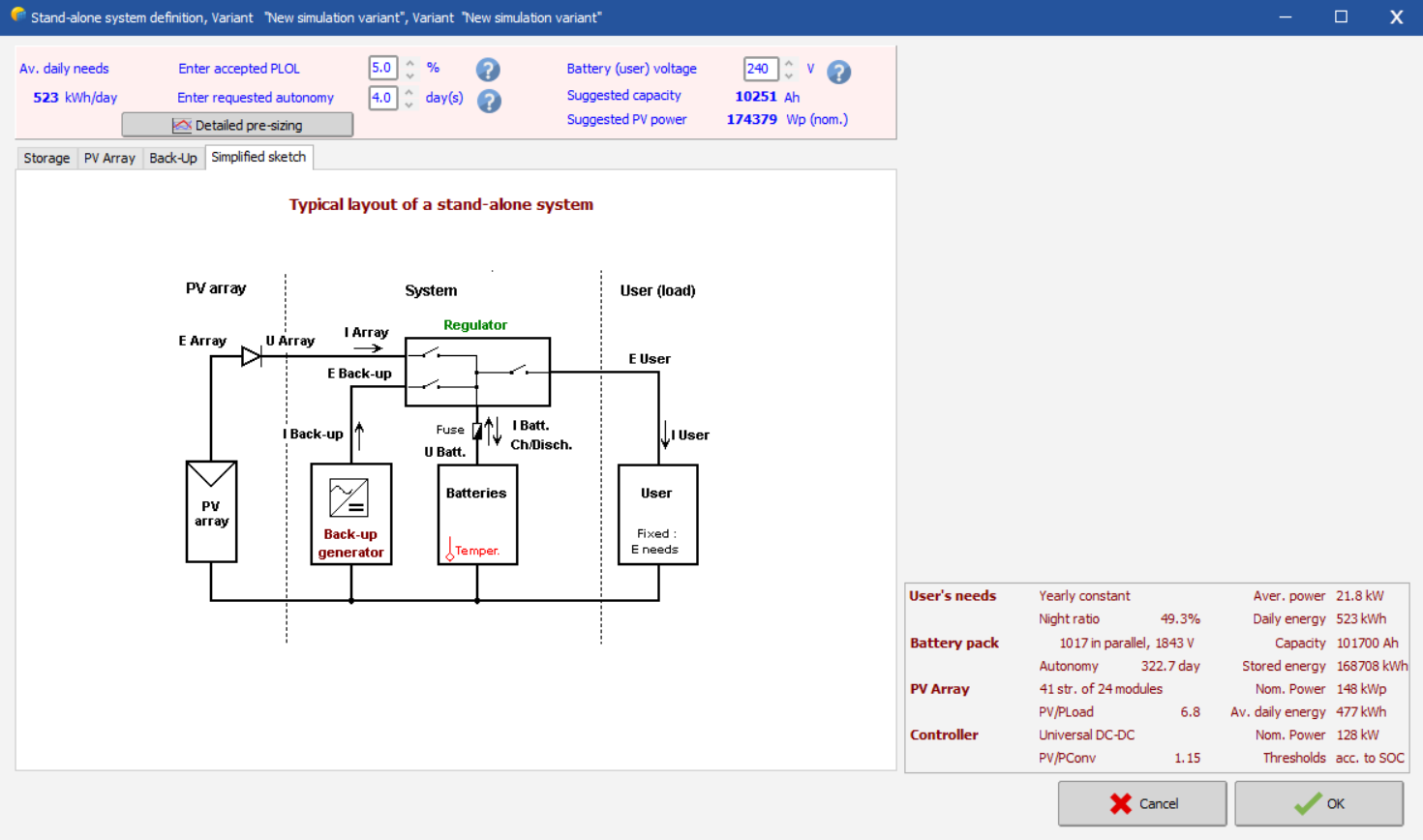


Figure S9 – Circuit diagram of the PV System

Figure S9 shows the circuit diagram of the stand-alone solar PV system of the business complex.


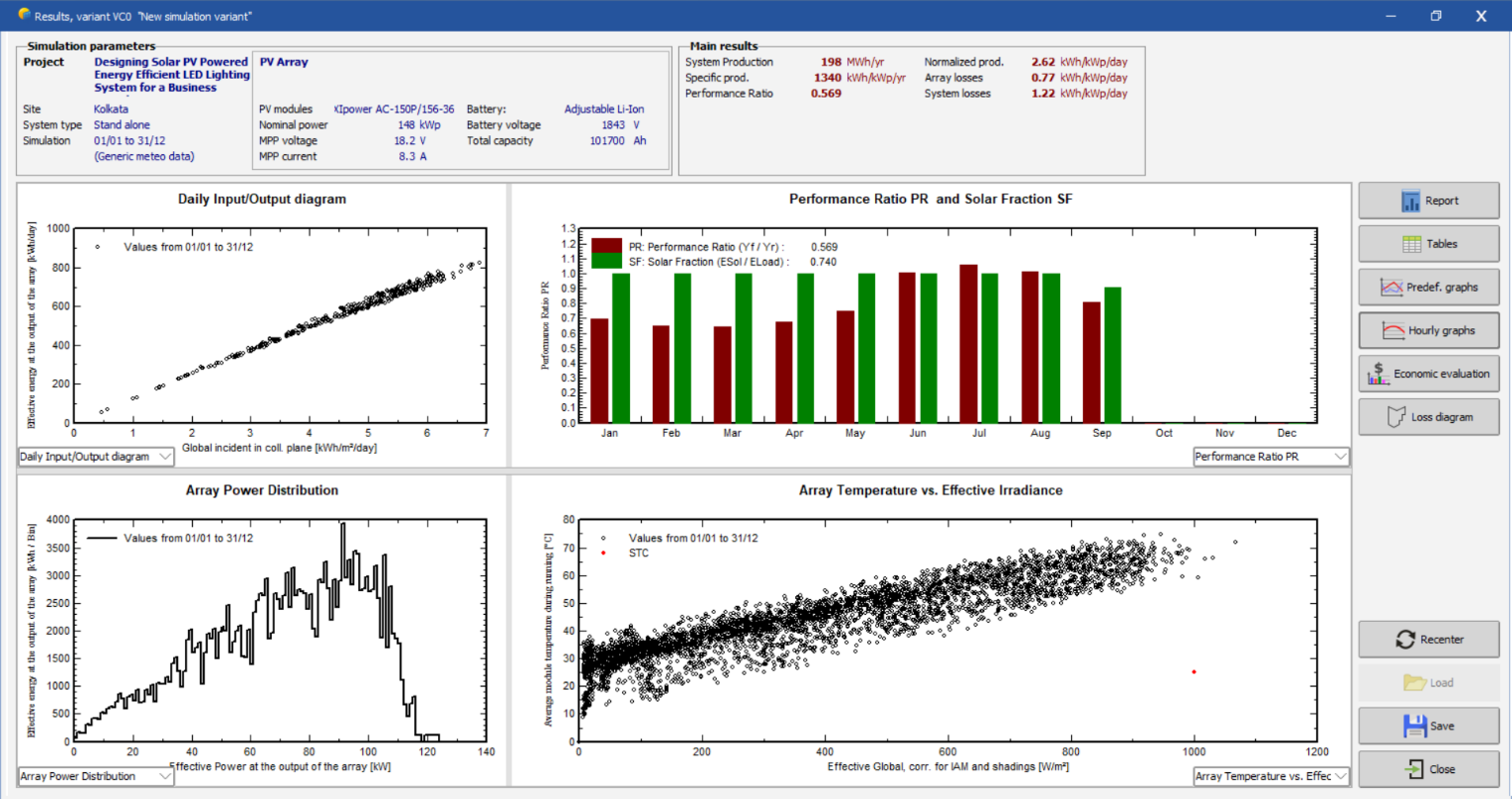


Figure S10 – Software generated report for Stand-alone PV system

Figure S10 shows the final software generated report for the stand-alone PV system. First considering the daily input/output diagram, the –X axis represents the global incident on collector plate in kWh/m^2^/day and the –Y axis represents the effective energy at the output of the array in kWh/day. It could be observed that the output energy increases with the increase in incident solar radiation.

The second graph is the performance ratio (PF) and solar fraction (SF) graph. Here, the –X axis represents the performance ratio (PF) and the –Y axis represents the time in months. The PF value got from the simulation is 0.569 and the solar fraction got is 0.740.

The third graph is the array power distribution. The –X axis represents the effective power at the output of the array in kW and the –Y axis represents the effective energy at the output of the array in kWh. The curve drawn here is for the entire year from 01/01/2021 to 30/12/2021.

The fourth graph is the array temperature versus effective irradiance. The –X axis represents the effective irradiance in W/m^2^ of the array in kW and the –Y axis represents the average module temperature during running in °C. It is estimated for the entire year from 01/01/2021 to 30/12/2021. From the graph it could be established that the temperature of the module increases with increase in solar irradiation but the performance decreases.

1. **Cost Estimation –**

For energy-producing organizations, each has their own cost per unit of electricity, with the interests of each organization in mind. However, here we will determine the unit rate without considering debits, capital and taxes. On considering our stand-alone solar PV plant for the business complex, the breakup of the capital cost will be

Total Load of the Business Complex = 522.824 Kilowatt-hours/day

Safety Factor = For safety we will be calculating the prices for double the load i.e., 1 MW.

Cost of a **Microtek 150W/12V Polycrystalline Panel =** ₹ 8,000

Total cost of solar panel = ₹ 4,88,16,000

Cost of inverter (500 kW) = ₹ 33,00,000

Cost of mounting structure = ₹ 31,36,944

Miscellaneous costs (cables and distribution boxes) = ₹ 2,87,55,320

Cost of installation = ₹ 4,44,40,040.

Therefore, the total cost would be ₹ 12,84,08,304.

1. **References –**

| [1] Unbound Solar. 2022. Home Appliances Power Consumption Table.  <https://unboundsolar.com/solar-information/power-table> |
| --- |
| [2] Building Services Operation and Maintenance Executives Society. <http://www.bsomes.org.hk/upload_pdf/GPRD2016_S1-4.pdf> |
| [3] Unbound Solar. 2022. Home Appliances Power Consumption Table.  <https://unboundsolar.com/solar-information/power-table> |
| [4] India Solar Resource, Global Horizontal Irradiance, Solar Energy Centre, National Renewable Energy Laboratory, Anthony Lopez, Billy Roberts: April 25, 2013.  <http://www.nrel.gov/international/ra_india.html> |
| [5] The Economic Times. 2019. Commonly used solar panels in India, their features and how to choose an ideal one as per your requirements.  <https://economictimes.indiatimes.com/small-biz/productline/power-generation/monocrystalline-vs-polycrystalline-solar-panel-which-is-most-suitable-for-power-requirements/articleshow/69202894.cms> |
| [6] Keisan Online Calculator, 2022 CASIO COMPUTER CO., LTD. <https://keisan.casio.com/exec/system/1224682277> |
